# Visualization of Brain Tumors with Infrared-Labeled Aptamers for Fluorescence-Guided Surgery

**DOI:** 10.1021/jacs.4c06716

**Published:** 2024-08-26

**Authors:** Galina Zamay, Anastasia Koshmanova, Andrey Narodov, Anton Gorbushin, Ivan Voronkovskii, Daniil Grek, Natalia Luzan, Olga Kolovskaya, Irina Shchugoreva, Polina Artyushenko, Yury Glazyrin, Victoriya Fedotovskaya, Olga Kuziakova, Dmitry Veprintsev, Kirill Belugin, Kirill Lukyanenko, Elena Nikolaeva, Andrey Kirichenko, Ivan Lapin, Vladimir Khorzhevskii, Evgeniy Semichev, Alexey Mohov, Daria Kirichenko, Nikolay Tokarev, Natalia Chanchikova, Alexey Krat, Ruslan Zukov, Varvara Bakhtina, Pavel Shnyakin, Pavel Shesternya, Felix Tomilin, Aleksandra Kosinova, Valery Svetlichnyi, Tatiana Zamay, Vadim Kumeiko, Vasily Mezko, Maxim V. Berezovski, Anna Kichkailo

**Affiliations:** † Prof. V.F. Voino-Yasenetsky Krasnoyarsk State Medical University, Krasnoyarsk 660022, Russia; ‡ Aptamerlab LLC, Krasnoyarsk 660042, Russia; § Federal Research Center “Krasnoyarsk Science Center of the Siberian Branch of the Russian Academy of Sciences”, Krasnoyarsk 660036, Russia; ∥ Krasnoyarsk Inter-District Ambulance Hospital Named after N.S. Karpovich, 17 Kurchatova, Krasnoyarsk 660062, Russia; ⊥ A.V. Zhirmunsky National Scientific Center of Marine Biology, Far Eastern Branch of Russian Academy of Sciences, Vladivostok 690041, Russia; # School of Medicine and Life Sciences, Far Eastern Federal University, Vladivostok 690922, Russia; ¶ Federal Siberian Research Clinical Centre under the Federal Medical Biological Agency, Krasnoyarsk 660130, Russia; ∇ Laboratory of Advanced Materials and Technology, Tomsk State University, Tomsk 634050, Russia; ○ Krasnoyarsk Regional Pathology-Anatomic Bureau, Partizana Zheleznyaka, Krasnoyarsk 660022, Russia; ⧫ Krasnoyarsk Regional Clinical Cancer Center, 16 1-ya Smolenskaya, Krasnoyarsk 660133, Russia; †† Kirensky Institute of Physics, Krasnoyarsk 660036, Russia; ‡‡ Department of Chemistry and Biomolecular Sciences, University of Ottawa, Ottawa, Ontario K1N 6N5, Canada

## Abstract

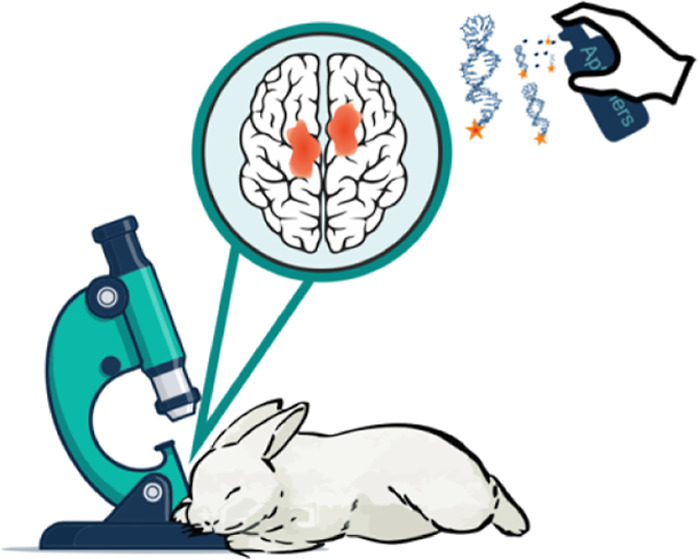

Gliomas remain challenging brain tumors to treat due to their infiltrative nature. Accurately identifying tumor boundaries during surgery is crucial for successful resection. This study introduces an innovative intraoperative visualization method utilizing surgical fluorescence microscopy to precisely locate tumor cell dissemination. Here, the focus is on the development of a novel contrasting agent (IR-Glint) for intraoperative visualization of human glial tumors comprising infrared-labeled Glint aptamers. The specificity of IR-Glint is assessed using flow cytometry and microscopy on primary cell cultures. In vivo effectiveness is studied on mouse and rabbit models, employing orthotopic xenotransplantation of human brain gliomas with various imaging techniques, including PET/CT, in vivo fluorescence visualization, confocal laser scanning, and surgical microscopy. The experiments validate the potential of IR-Glint for the intraoperative visualization of gliomas using infrared imaging. IR-Glint penetrates the blood–brain barrier and can be used for both intravenous and surface applications, allowing clear visualization of the tumor. The surface application directly to the brain reduces the dosage required and mitigates potential toxic effects on the patient. The research shows the potential of infrared dye-labeled aptamers for accurately visualizing glial tumors during brain surgery. This novel aptamer-assisted fluorescence-guided surgery (AptaFGS) may pave the way for future advancements in the field of neurosurgery.

## 1 Introduction

Gliomas are malignant primary brain tumors that primarily occur in adults. By cell origin, they are currently classified as astrocytomas (grades 2–4), oligodendrogliomas (grades 2–3), and IDH1-wildtype glioblastoma (grade 4) (GB).^1^ The latter is considered the most common and aggressive. GB exhibits substantial heterogeneity at the cytopathological, transcriptomic, and genomic levels. Its aggressive nature is attributed to uncontrolled cellular proliferation, resistance to programmed cell death (apoptosis), heightened angiogenesis, progressive infiltration into the surrounding healthy brain tissue, and genomic instability.

Neurosurgery is one of the critical steps in glioma therapy; its subtotal resection is highly important. Accurate delineation of the true tumor boundaries is challenging due to its diffusely infiltrative nature. Moreover, the extent of brain involvement, as visualized through contrast enhancement, merely represents a macroscopic view and does not reveal the complete magnitude of tumor invasion.^2^ To enhance patient survival rates and minimize tumor recurrence, it is imperative to meticulously excise all malignant cells dispersed within healthy tissue, distant from the primary tumor focus during surgical interventions.

Several methods of intraoperative visualization have been proposed to accurately determine the localization of tumor cells, which can provide real-time visualization of neoplasms using specific or nonspecific fluorescent dyes and a surgical fluorescent microscope. One of the most commonly used substances that can passively accumulate in tumor tissue is indocyanine green (760–820 nm), which has been utilized in neurosurgery since 2003 for intraoperative assessment of aneurysms, arteriovenous malformations, and cortical perfusion.^3^ Despite their wide application and proven effectiveness, drugs that passively accumulate in tissues have significant drawbacks,^4^ such as

(1) The nonspecific cellular uptake of the drug can result in areas of tissue with increased metabolism (e.g., inflammatory foci, edema zones) emitting fluorescence similar to that of tumor tissue.
(2) Due to the angiogenesis and vascularization of solid tumors, which lead to the proliferation and entanglement of blood vessels, the access of the drug to cancer cells may be hindered, resulting in them remaining unstained.

To overcome the aforementioned issues, nonspecific fluorophores should be conjugated with tumor-specific ligands such as antibodies, peptides, or aptamers.^4,5^ Analysis of the scientific literature and products undergoing preclinical and clinical trials has revealed a wide array of agents for intraoperative tumor visualization based on infrared (IR) fluorophores conjugated with monoclonal antibodies. One such agent is Cetuximab-IRDye800CW (NCT02855086), which combines the IR dye IRDye800CW, recommended by the Food and Drug Administration (FDA), with an antibody against the human epidermal growth factor receptor (EGFR).

EGFR/ErbB-1/HER1 is a transmembrane protein belonging to the ErbB receptor family, increased expression of which is observed in various types of cancers such as glioblastoma, nonsmall-cell lung cancer (NSCLC), pancreatic ductal adenocarcinoma, breast cancer, and head and neck squamous cell carcinoma (HNSCC).^6^ Cetuximab-800CW has been tested on nine HNSCC patients (NCT01987375) and is currently in phase I/II clinical trials for pancreatic ductal adenocarcinoma, malignant gliomas, and HNSCC (NCT02736578, NCT02855086, and NCT03134846). One more drug based on an antibody to EGFR and IRDye800CW is Panitumumab-IRDye800 (NCT03510208). ABX-EGF Monoclonal Antibody binds EGFR with high affinity (5 × 10^–11^ M),^7^ blocks the binding of both EGF and transforming growth factor-α (TGF-α) to various EGFR-expressing human carcinoma cell lines, and inhibits EGF-dependent tumor cell activation. Another IR fluorophore, BLZ-100, based on a chlorotoxin peptide specific to annexin A2, is in the early stages of clinical trials.^8^

The key advantage of monoclonal antibodies is their high specificity toward target cells, minimizing damage to healthy cells and resulting in fewer side effects compared to traditional small organic molecule-based drugs. However, antibodies also have some drawbacks.^9^ For instance, the crystallizable fragment of antibodies can interact with Fc receptors expressed on the surface of various cell types, increasing their cross-reactivity and promoting retention in the bloodstream. Other limitations are associated with the complexity of production, as therapeutic antibodies require large-scale mammalian cell culture and subsequent stringent purification under good manufacturing practices. Furthermore, monoclonal antibodies developed in animals need to be specially prepared for administration into the human body in clinical settings.^10^ In addition, antibodies have a short shelf life, and although they can be chemically modified, site-specific modifications are extremely difficult.^11,12^

These constraints have led to the development of aptamers that are characterized by ease of production, chemical synthesis, and modification. The aptamers, small (5–30 kDa) single-stranded DNA or RNA molecules, carry a nucleotide-based code in their primary sequence, allowing for easy synthesis and modification. They fold into unique three-dimensional structures, exhibit high affinity for targets, and can be used for inhibiting or activating specific proteins. Aptamers are often referred to as “synthetic antibodies”, but in many aspects, they surpass antibodies in the following aspects:^13^

(1) Synthesis costs of aptamers are 1000 times lower than obtaining antibodies.
(2) Aptamers are easily synthesized and modified.
(3) Aptamers are low immunogenic and low toxic.
(4) Small size allows better tissue penetration and clearance from the body.

Aptamers offer a promising alternative to antibodies with advantages in terms of synthesis, modification, immunogenicity, and toxicity, making them a valuable tool for various applications in biotechnology and medicine. Currently, specific near-infrared dyes based on antibodies and peptides are emerging for the intraoperative diagnosis of glial tumors. However, today, no aptamer-based contrasting agent is still available worldwide for the intraoperative visualization of glioma.

Nowadays, diverse aptamers specific to glial tumor markers have been selected. For instance, a systematic review by Nuzzo et al. in 2020 describes thirty-eight aptamers, 17 of which have already been used in diagnostic studies, and twenty-one for glioblastoma therapy.^14^ Among them are DNA and RNA aptamers specific to nucleolin, EGFR, VEGF, PDGFR, and Tenascin-C.

However, aptamer usage in clinical practice is still limited by its sensitivity to nucleases in body fluids and the likelihood of not penetrating the blood–brain barrier (BBB). These issues can be solved through chemical modifications and the selection of aptamers with the ability to overcome the BBB. Another solution to the problem of aptamer penetration through the BBB and their conjugates can be their direct application to the tumor site during surgery.

Although aptamers are attractive for targeted therapy, they have not yet been used for intraoperative tumor staining. In this study, we have, for the first time, developed a contrasting agent based on DNA aptamers, named IR-Glint, and a commercially available infrared fluorophore Cy7.5 for intraoperative visualization of glioma and Aptamer-assisted Fluorescence-Guided Surgery (AptaFGS).

IR-Glint demonstrates a high tumor-to-background ratio (TBR), does not exert any toxic effects on mice, and is eliminated through renal filtration and biliary excretion.

## 2 Results

### 2.1 Modification of Gli-233 and Gli-55 Aptamers

**Figure 1:**
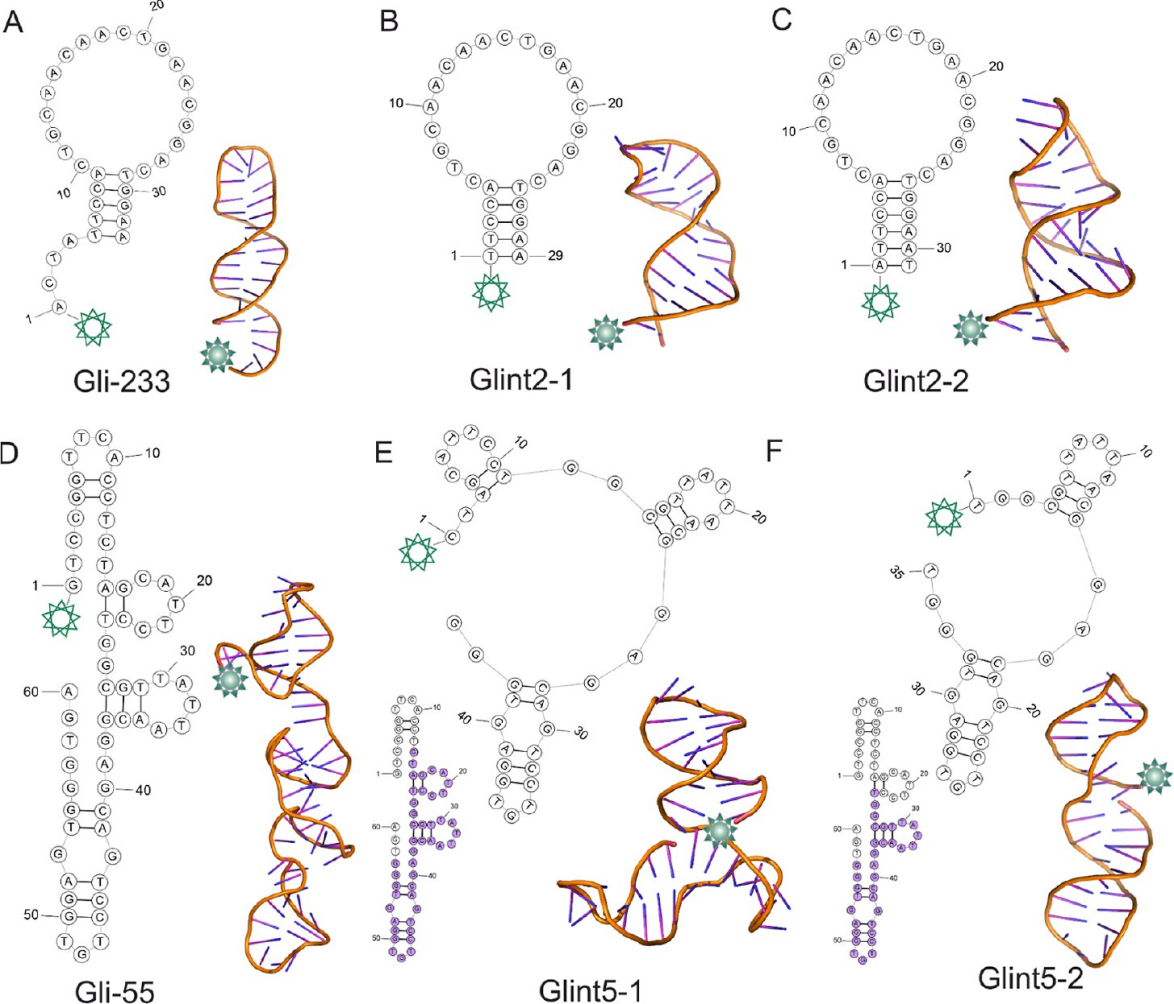
Secondary and tertiary structures of Gli-233 (A), Glint2-1 (B), Glint2-2 (C), Gli-55 (D), Glint5-1 (E), and Glint5-2 (F) aptamers. The green star indicates a fluorescent label attached at the 5′-end of aptamers. The purple color shows what nucleotides of the original aptamer are included in the modified ones.

Previously, we reported Gli-233 and Gli-55 aptamers that selectively bind to glial tumor cells.^5^ According to the mass spectrometry analyses, these aptamers have different binding partners, which is why, in order to increase the intensity of the fluorescent signal during surgery, both of them were chosen for developing a new contrasting agent. Originally, these aptamers were selected from the 100 nt Harvard library^15^ against glial tumor tissues taken after the surgical resection. The aptamers were truncated. The secondary and tertiary structures have been found by using the combination of small-angle X-ray scattering (SAXS) and molecular modeling. Gli-233 has a “hairpin” shape with a rather big single-stranded loop maintained by five base pairs (Figure 1A). Considering the size of the loop and helix parts, four nucleotides at the 5′ end were considered nonessential for both binding and structure-maintaining functions. Moreover, the more nucleotides, the more options for aptamer folding. In order to avoid the formation of other undesirable structures of Gli-233, two modified options of the aptamer were proposed. The single-stranded part at the 5′ end was removed, and one base pair was added to enhance the stability of the aptamer structure in the solution. Thus, the resulting two aptamers, Glint2-1 and Glint2-2, were modeled. The secondary and tertiary structures of the aptamers are presented in Figure 1B,C.

Aptamer Gli-55 consists of 60 nucleotides (Figure 1D). Thus, there is a need to reduce its size, considering the cost of the synthesis and the future possibility of using this aptamer as a part of a new bivalent or multivalent aptamer. First, the aptamer Gli-55 had 13 nucleotides removed from the 5′ end. The aim of the truncation was to remove a free strand of nucleotides, consisting of 4 nucleotides, that would not be able to provide binding specificity with the protein target. However, removing this strand would have led to the disruption of a short double-stranded region, which would have resulted in the formation of a longer single-stranded segment. Therefore, the first hairpin at the 5′-end of aptamer Gli-55 was completely removed from the structure, while three stem-loops at the 3′ end remained unchanged compared to the original aptamer Gli-55 (Figure 1E). Then, the next stem-loop from the 5′ end was truncated to simplify the structure. According to Figure 1F, two stem-loops at the 3′ end also remain unchanged. Thus, two truncated sequences of Gli-55 with three hairpins (Glint5-1) and two hairpins (Glint5-2) were modeled (Figure 1E,F).

For all new aptamers, molecular dynamics (MD) simulations were carried out to imitate the in vitro environment: solvent, temperature, and presence of the ions. Secondary and tertiary structures of the aptamers obtained as a result of clustering analysis of 200 ns MD trajectories are shown in Figure 1.

### 2.2 Molecular Profiling of Primary Cell Cultures

**Figure 2:**
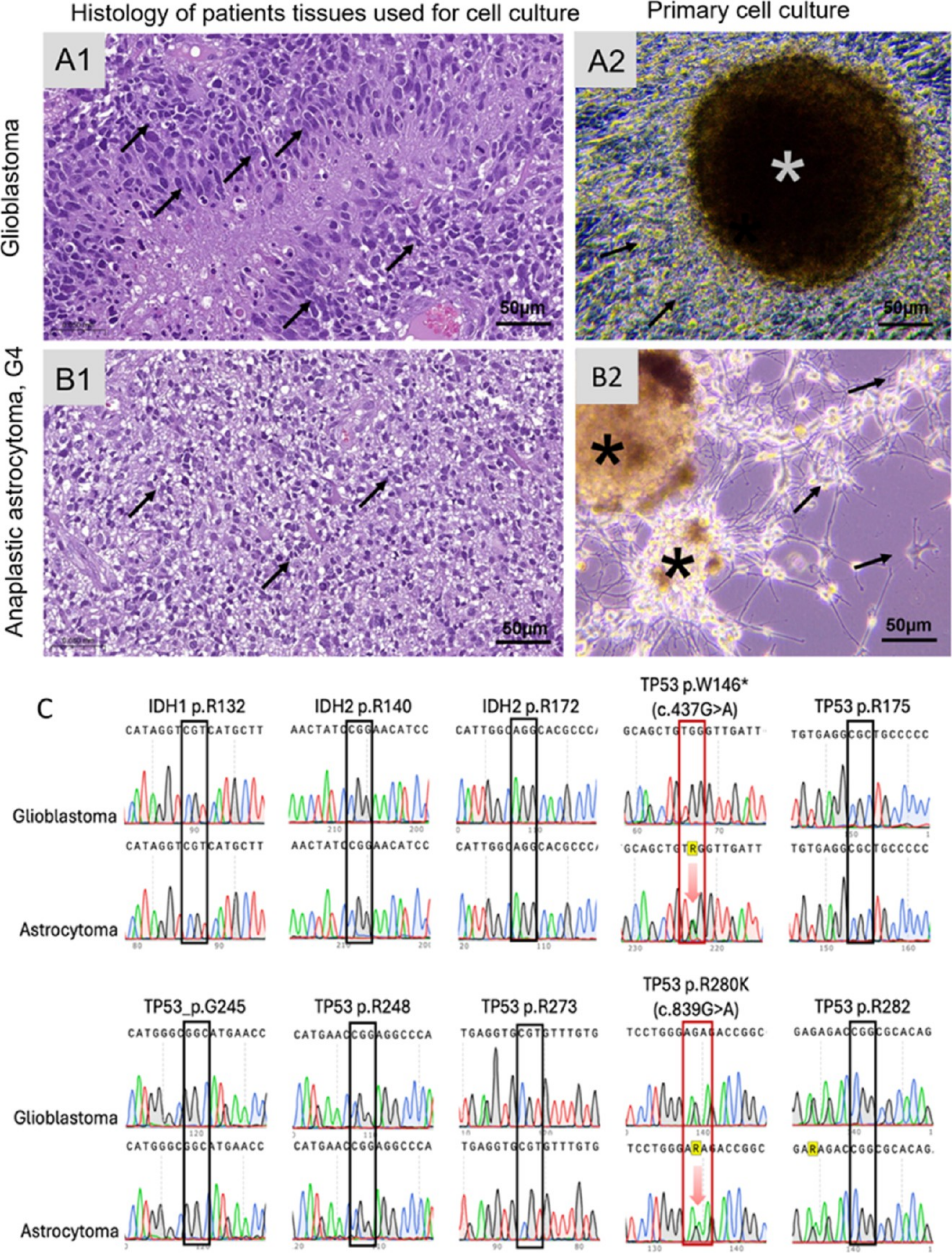
Primary cell culture characterization. (A) Morphology of glioblastoma (A1) and anaplastic astrocytoma G4 (B1) tissues used for the primary cell culture establishment, the sections stained with hematoxylin and eosin (H&E). Primary cell cutlers from glioblastoma (A2) and astrocytoma (B2), containing cells attached to the bottom of the well (black arrows) and oncospheres (*). Sanger sequencing results for glioblastoma and astrocytoma (C). Two mutations in TP53 (shown as red arrows in red rectangles) were discovered in the cells of the human astrocytoma model in a heterozygous state.

Two primary cultures with high proliferation activity were used to estimate the utility of IR-Glint on xenotransplanted glial tumor models. Primary cell cultures were evolved from surgically resected and histologically verified glioblastoma (Figure 2A1) and anaplastic astrocytoma G4 (Figure 2B1). Cell cultures contain disseminating cells and oncospheres (Figure 2A2,B2). Molecular profiling was performed to gain a deeper understanding of the nature of the tumors. Hotspots in IDH1, IDH2, and TP53 were identified using Sanger sequencing. These specific genes were selected because they provide valuable information about glioma proliferation and migration patterns. Mutations in IDH1 and IDH2 are associated with higher overall survival rates and are more commonly found in low-grade gliomas (LGG). TP53 mutations, on the other hand, play a role in the transformation from LGG to high-grade gliomas (HGG). According to data from The Cancer Genome Atlas (TCGA) and recent publications, pathogenic mutations in TP53 are frequently observed in glioblastomas but are far less common in LGG. These mutations are also associated with poor survival rates in patients.^16^

Sanger sequencing analysis revealed that the astrocytoma used in the human astrocytoma model harbors two heterozygous mutations in TP53: p.W146* (c.437G > A), which leads to a loss of p53 protein function, and p.R280K, which results in increased cell migration and invasion and increased cell survival.^17,18^ This is a reason for the increase in the astrocytoma’s degree up to grade 4. In contrast, glioblastoma does not carry mutations in the studied areas (Figure 2C).

The primary cell cultures were used for flow cytometry analyses and animal models of glial tumors.

### 2.3 Flow Cytometric Aptamer Affinity Analysis

**Figure 3:**
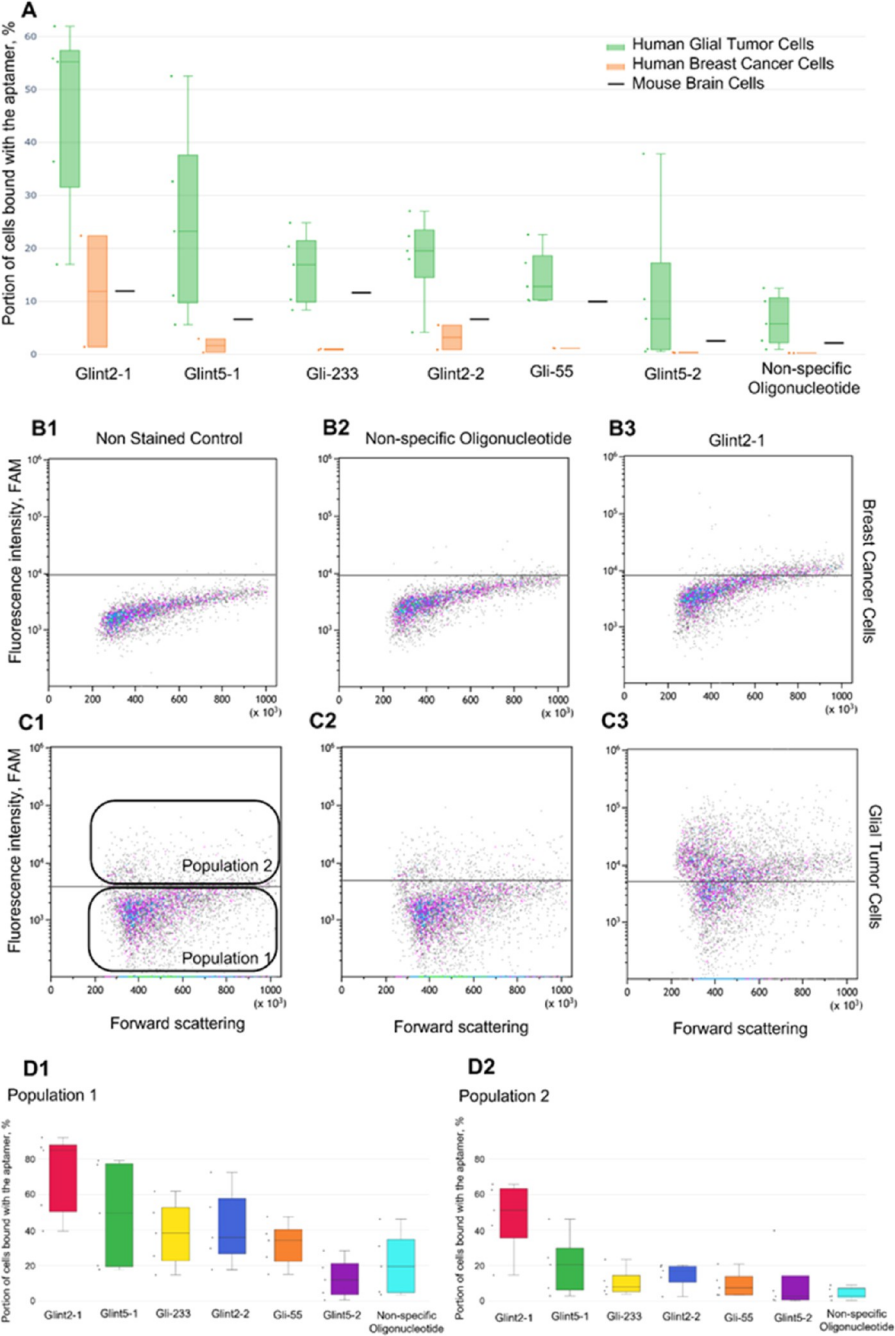
Binding analysis of aptamers to cells. Histogram of aptamers binding with human glial tumor cells and control cells: human breast cancer cultured cells and cells isolated from healthy mouse brain (A). Flow cytometry dot plots of human breast cancer (B) and human glioblastoma (C) cells: nonstained control (1), incubation with FAM-labeled nonspecific randomized oligonucleotide (2), and Glint2-1 (3). Histograms of aptamer binding with population 1 (D1) and population 2 (D2) of primary cultured human glioblastoma cells (D).

The binding of Gli-233, Glint2-1, Glint2-2, Gli-55, Glint5-1, and Glint5-2 aptamers was evaluated by using flow cytometry. Primary glial tumor cell cultures derived from tissues of 5 different patients, primary breast cancer cell cultures derived from 2 different patients, and cells isolated from the brain of a healthy mouse were used to estimate the binding affinity and specificity of the aptamers (Figure 3). Modified aptamers Glint2-1 and Glint5-1 showed the best binding to primary human glial tumor cell culture compared with the initial aptamers, other modified sequences, and 40 nt nonspecific randomized oligonucleotides. Glint2-1 and Glint5-1 demonstrated much lower binding with controls such as human breast cancer cells and cells isolated from the brain of a healthy mouse (Figure 3A).

Figure 3C,D represents dot plot histograms obtained for a nonspecific oligonucleotide and Glint2-1, compared with a nonspecific randomized oligonucleotide used as a control in binding experiments for human glial tumor cells and for breast cancer cells. The primary glial cell culture consisted of two cell types (Figure 2B2): cells forming oncospheres (Population 1) and adherent cells (Population 2) (depicted in Figure 3C,D). Population 2 initially exhibited a higher level of autofluorescence and demonstrated a lower binding rate (Figure 3D).

The obtained data show that compared to the other aptamers, Glint2-1 and Glint5-1 aptamers exhibited a higher binding percentage to human glioma cells and a lower binding percentage to human breast cancer cells and cells isolated from the brain of a healthy mouse. Glint2-1 and Glint5-1 were specific to glial tumor cells and thus have been chosen for further experiments and the development of a Cy 7.5-based spray for intraoperative staining.

### 2.4 Evaluation of Aptamer Binding Ex Vivo

**Figure 4:**
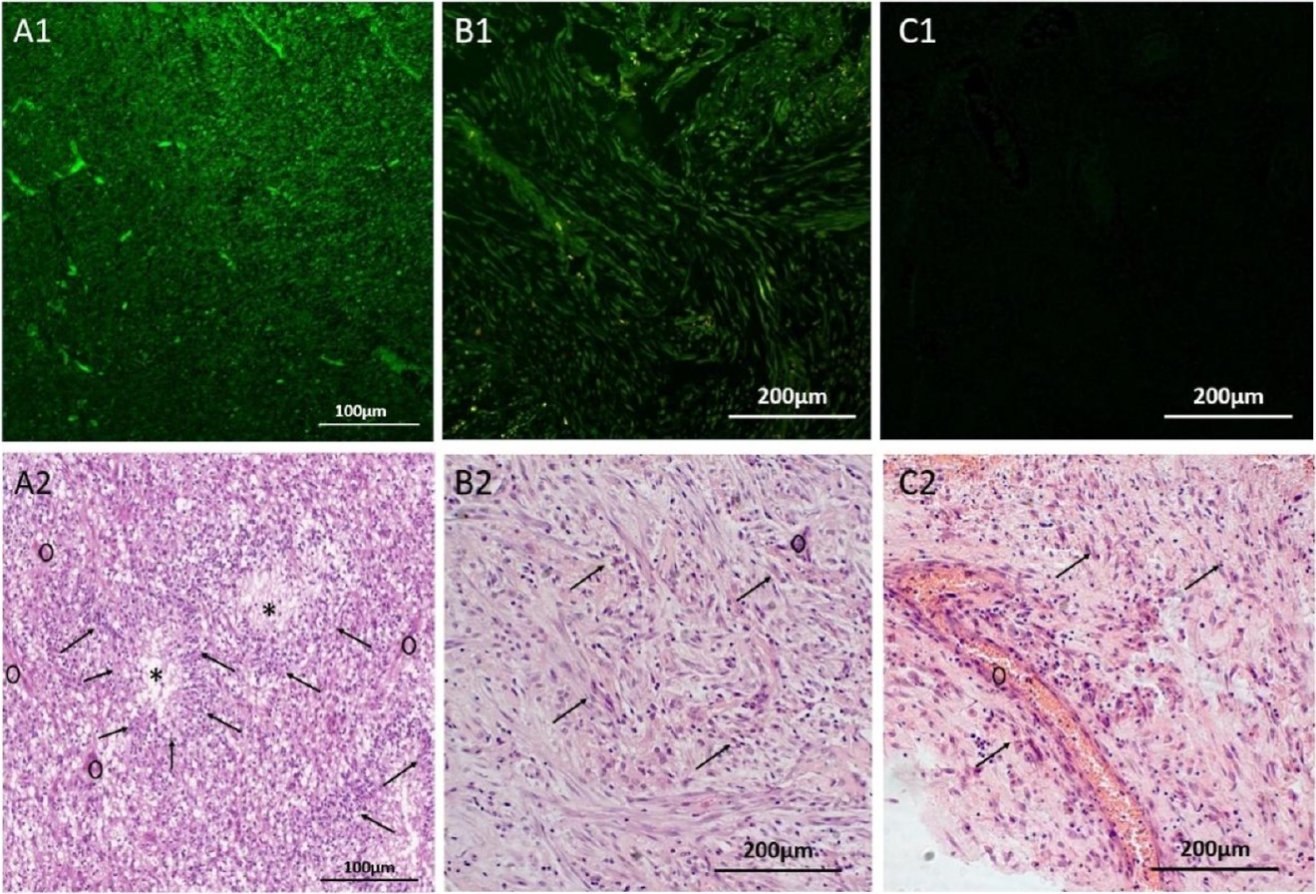
Specific staining of glial tumor cells in surgically resected glioblastoma tissues. In panels, (A1,B1) FAM-Glint was used to stain glioblastoma multiform (A) and sarcomatoid region in glial tumor (B). Panel (C) shows the staining of glial tumor tissues obtained with a FAM-labeled 40 nt nonspecific randomized oligonucleotide (C1). Adjacent sections stained with hematoxylin and eosin (H&E) were used for comparison in panels (A2–C2). Arrows indicate glial tumor cell clusters; *—necrotic areas and black circles—blood vessels.

FAM-labeled Glint2-1 and Glint5-1, at concentrations of 50 nM each, were pooled together (FAM-Glint) and used to stain the tissues ex vivo. Histological analysis demonstrated that FAM-Glint indeed stained glial tumors (Figure 4A,B), compared to the FAM-labeled 40 nt nonspecific randomized oligonucleotide, which did not stain (Figure 4C) the glial tumor cells (black arrows in Hematoxylin and eosin (H&E) adjacent section) and blood vessels (black circles in H&E adjacent section). The aptamers specifically bind to glial tumor cells (black arrows in H&E adjacent section), as shown by the distinct staining pattern of the glioblastoma multiforme region (Figure 4A), as well as the tumor cells (black arrows in H&E adjacent section) in the sarcomatoid region of the glial tumor (Figure 4B), which can be clearly observed. Overall, these results demonstrate the specific binding capabilities of the aptamers to human glial tumor cells in glioblastoma tissues regardless of their histological features, as confirmed by both FAM-Glint staining and H&E analysis. This concentration was sufficient for confocal microscopy; however, for ex vivo and in vivo tissue staining, the concentration was optimized.

**Figure 5:**
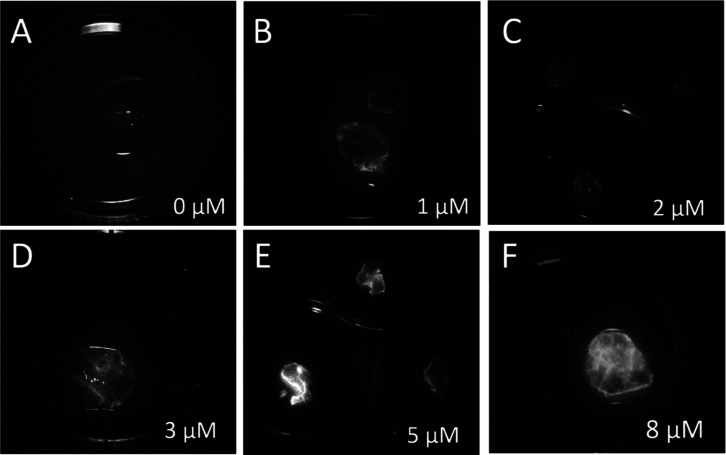
Glial tumor samples were incubated with a 1:1 mixture of Cy7.5 labeled Glint2-1 and Glint5-1 aptamers at concentrations of 0 (A), 1 (B), 2 (C), 3 (D), 5 (E), and 8 μM (F) using the IR module of the Zeiss Kinevo 900 surgical microscope. The tissues were placed in 3 cm Petri dishes.

The optimal concentration of the IR-Glint for fluorescent-guided surgery was evaluated on-site using a Fluor i In Vivo imaging system (South Korea) with a red excitation laser, an infrared-emitting filter, and a Zeiss Kinevo 900 fluorescent surgical microscope (Carl Zeiss, Germany). Figure S1A shows IR-Glint at 0, 1, and 2 μM in a tube, which was diluted in DPBS. As seen in the images, both concentrations are visible under the IR fluorescent module of the surgical microscope. To determine the optimal concentration for specific visual guidance during tumor surgery, freshly resected glioma tissues were stained with individual Cy7.5-labeled Glint2-1 and Glint5-1 (Figure S1B) or a 1:1 mixture of Cy7.5-labeled Glint2-1 and Glint5-1 called IR-Glint at various concentrations (Figures 5 and S1C). Using the fluorescence imaging system, we demonstrated that IR-Glint is capable of staining similar glial tissue samples in a reproducible and dose-dependent manner (Figure S1C). The mixture of the scrambled sequences of Glint2-1 and Glint5-1 did not bind to a glial tumor, while IR-Glint stained a glial tumor well (Figure S1D). The best imaging using an infrared-emitting filter on the fluorescent surgical microscope Zeiss Kinevo 900 (Carl Zeiss, Germany) with the infrared module IR800 was performed with 5 μM IR-Glint (Figure 5).

### 2.5 Simulation of Aptamer-Assisted Fluorescence-Guided Surgery in Mice

The main goal of the AptaFGS simulation experiment was to choose a strategy for administering the IR-Glint: intravenous injection or surface application. The surface application reduces aptamer dose usage, potential toxic effects on the patient, and the decreased risk of oligonucleotide degradation in the blood due to nucleases. Intravenous administration allows for staining of the entire tumor site just before surgery, which can assist the surgeon in removing the tumor. However, there is a risk that the aptamer may not cross the blood–brain barrier.

We used healthy mice and mice with human orthotopically transplanted glioma to determine the optimal strategy for administering the IR-Glint, or nonspecific oligonucleotide or indocyanine green, a contrasting agent used in clinics. Fluorescence of IR-Glint in mice was evaluated using the Fluor i In Vivo imaging system (South Korea).

Before intravenous administration of IR-Glint, healthy mice and noninjected mice with transplanted tumors in the brain showed no background infrared fluorescence (Figures S2A and S4). Five minutes after the tail vein injection, the bodies of the mice also did not emit fluorescent light (Figure S2B). However, after 40 min, IR-Glint distributed all around the body (Figure S2C). Ninety minutes after injection, the dye became visible in the abdominal cavity (Figure S2D), and it can be seen that IR-Glint accumulates in the liver, gallbladder, kidneys, and intestines (Figure S2F) 5 h after administration. Residual fluorescence was visualized after 24 h, only in the tail in case a vein bursts during injection (Figure S2E).

In healthy mice with the trepanation hole of the skull, intracranially applied IR-Glint was visualized in the head at the site of inoculation for at least 90 min (Figure S3A–C). It is also metabolized in the liver and spleen, filtered through the kidneys, and excreted through the intestines. After 2 h, it can still be seen at the site of its application (Figure S3D).

**Figure 6:**
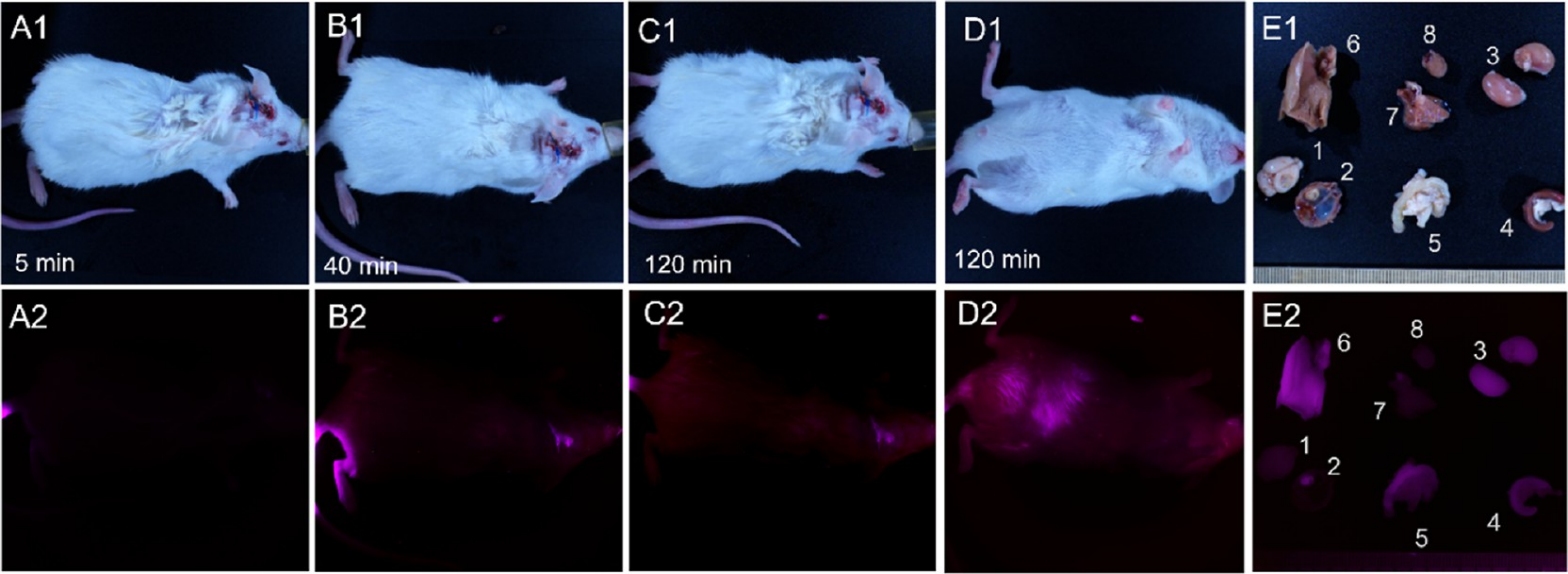
Distribution of IR-Glint in mice after tail vein injection registered at Brightfield (1) and IR fluorescence (2). Mouse after 5 (A), 40 (B), and 90 min (C,D) of tail vein injection. Accumulation of IR-Glint in organs (E) 1—brain, 2—tumor inside the skull, 3—kidneys, 4—spleen, 5—intestines, 6—liver, 7—lungs, and 8—heart.

**Figure 7:**
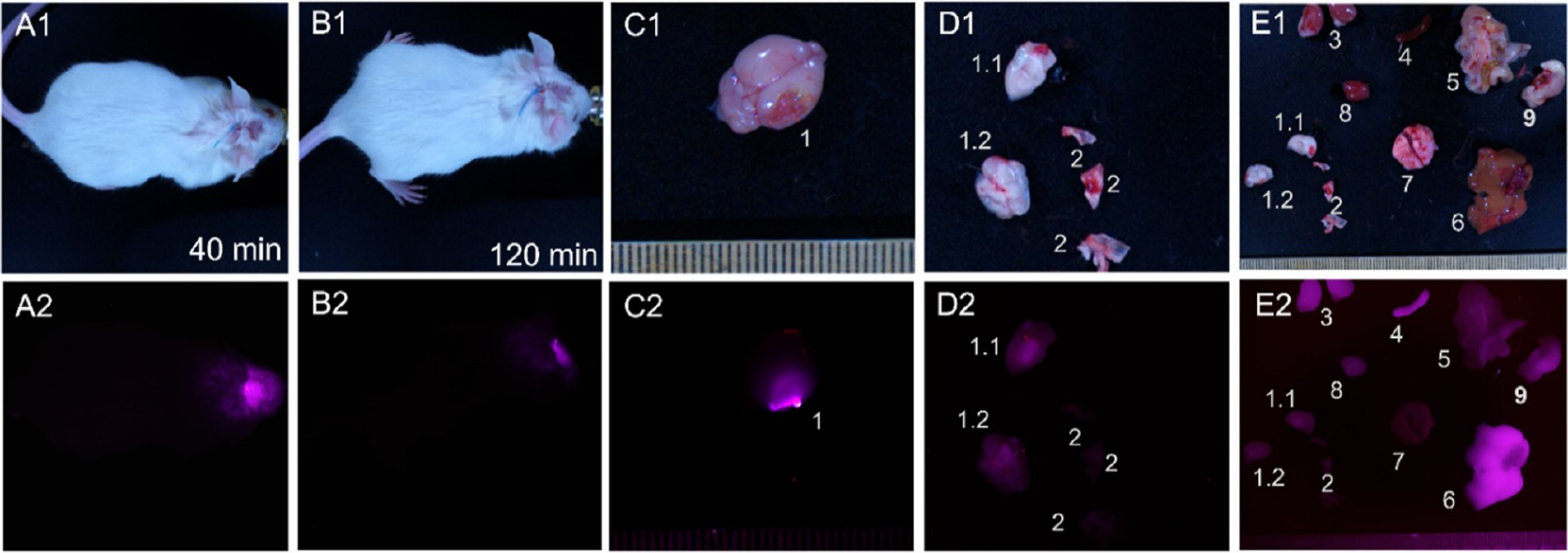
Distribution of IR-Glint in mice after intracranial injection (subcutaneously on the place of trepanation hole) registered at Brightfield (1) and IR fluorescence (2). Mouse after 40 (A) and 120 min (B) of subcutaneous injection. Accumulation of IR-Glint in the brain (C) and dissected brain (D), comparing with other organs (E) 1.1, 1.2—dissected brain, 2—tumor inside the skull, 3—kidneys, 4—spleen, 5—intestines, 6—liver, 7—lungs, 8—heart, and 9—stomach.

Mice with orthotopically xenotransplanted gliomas exhibited a similar distribution of this contrasting agent when administered intravenously (Figure 6) and intracranially (Figure 7). 40 minutes after tail vein injection, IR-Glint could be visualized in the infrared spectra in the brain at the location where the tumor was transplanted. After 90 min, the tumor remained visible and became visible in the abdominal cavity (Figure 6D2). It could be observed that IR-Glint accumulated in the brain tumor, liver, gallbladder, kidneys, and intestines (Figures 6E2 and 7E2).

Cy7.5-labeled nonspecific oligonucleotides did not accumulate in the brain tumor regardless of where it was administered (Figure S5).

The commercially available tumor contrast agent indocyanine green, injected intraperitoneally 2 h before visualization, showed similar results and accumulated in the transplanted glial tumor (Figure S6B). The mice skin with background fluorescence in the same wavelengths as indocyanine green did not allow in vivo imaging (Figure S6A).

The experiments described above were conducted using an in vivo imaging system; however, it is essential to evaluate the potential application of the same technique for visualization under a surgical microscope. We simulated the fluorescence-guided surgery (FGS) procedure by directly applying IR-Glint to the exposed brain, followed by two subsequent brain rinses after a 2 min interval. After a 30 min period following the manipulation, the mice were sacrificed, the brains and organs fixed with formalin, and further examined using a surgical microscope equipped with an infrared module.

The mouse brain with orthotopically xenotransplanted glioma, tumor inside the skull, and organs did not show any background IR fluorescence without IR-Glint administration (Figure S7).

**Figure 8:**
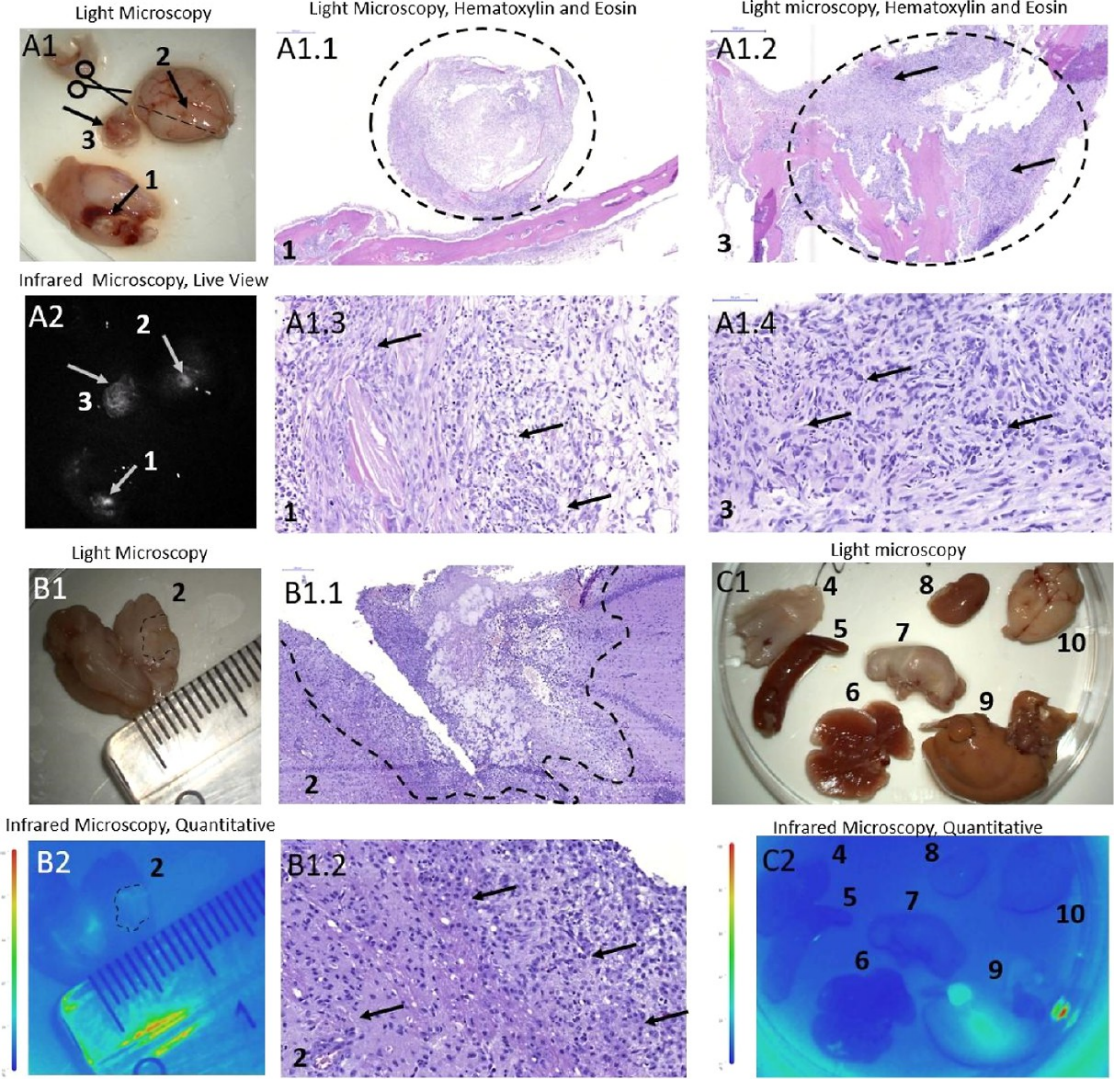
Light (A1–C1) and IR fluorescent (A2) microscopy of mouse brain (A, arrow 2), a tumor on the skull (A, arrow 1), a tumor on the skin (A, arrow 3), brain cross-section (B, dashed line indicates tumor area), and organs (C) with orthotopically xenotransplanted glioma after the surface intracranial application of IR-Glint. H&E staining confirmed the formation of astrocytoma G4 originated from glioblastoma in mice brains (A1.1–A1.4) and inside the skull (B1.1,B1.2). Dashed lines indicate the tumor, and arrows—represent tumor cell clusters. Panel (C) 4—skin, 5—spleen, 6—lungs, 7—stomach, 8—kidney, 9—liver, and 10—brain.

Figure 8 presents the brains and organs of mice subjected to FGS with surface-applied aptamers (Figure 8A) and intravenous injection (Figure 8B). As seen in the figures, the surface application of IR-Glint is sufficient for visualization under the surgical microscope with an IR module. It allowed for the visualization of tumor sites (Figure 8A2) with good contrast. Additionally, in cross-section, it can be observed that the IR-Glint penetrated 3–4 mm into the brain within 3 min. It also accumulated in the liver (Figure 8C2).

### 2.6 Toxicity Testing of Aptamers in Mice

The acute toxicity of IR-Glint was assessed by monitoring changes in various blood biochemical parameters, including the total protein level, cholesterol level, bilirubin level, alanine aminotransferase activity, alkaline phosphatase activity, and alpha-amylase activity. These specific parameters were chosen as they provide insights into the functionality of crucial organs, such as the pancreas, kidneys, and liver.

The total protein level indicates protein metabolism within the body, reflecting the overall health of the liver and kidneys. By measuring cholesterol levels, we can gain information about lipid metabolism and assess potential effects on cardiovascular health. Bilirubin, a bile pigment produced during the breakdown of heme-containing proteins such as hemoglobin, myoglobin, and cytochrome, is useful in determining the extent of red blood cell destruction and impaired bilirubin excretion, such as in cases of hemolytic jaundice (Figure S8).

Moreover, the activities of alanine aminotransferase, alkaline phosphatase, and alpha-amylase were monitored. Alanine aminotransferase is an enzyme primarily found in the liver, and changes in its activity may indicate liver damage or dysfunction. Alkaline phosphatase is an enzyme produced by the liver, bones, and other tissues, and its altered activity can signify liver- or bone-related disorders. Alpha-amylase, on the other hand, is a digestive enzyme mainly secreted by the pancreas and salivary glands, with smaller amounts present in other tissues. Changes in alpha-amylase activity can provide insights into poisoning, pancreatic and salivary gland issues, and renal insufficiency. By evaluating these blood biochemical parameters, we can assess the potential toxicity of IR-Glint and its impact on various vital organs and metabolic processes (Figure S8).

Alanine aminotransferase is a cytosolic enzyme found in hepatocytes, and an increase in its activity indicates liver cell damage. Alkaline phosphatase is an enzyme present in almost all tissues of the body, with a predominant localization in the liver, bones, and placenta. Total alkaline phosphatase activity increases when liver tissue, bone, or kidney damage occurs. Cholesterol is an important metabolite synthesized in the liver and is involved in the production of hormones, bile acids, and vitamin D. It also regulates the cell membrane permeability. The concentration of cholesterol reflects the liver’s condition and impacts a wide range of metabolic pathways (Figure S8). Studies have demonstrated that all investigated blood serum biochemical parameters in mice in the control and experimental groups were within the normal range (Figure S8).^19^

Therefore, it has been shown that IR-Glint does not exert acute toxic effects on the mouse organism and can simplify the tumor resection procedure for the surgeon.

### 2.7 In Vivo Visualization of Human Xenotransplantated Glioma and AptaFGS in a Rabbit

**Figure 9:**
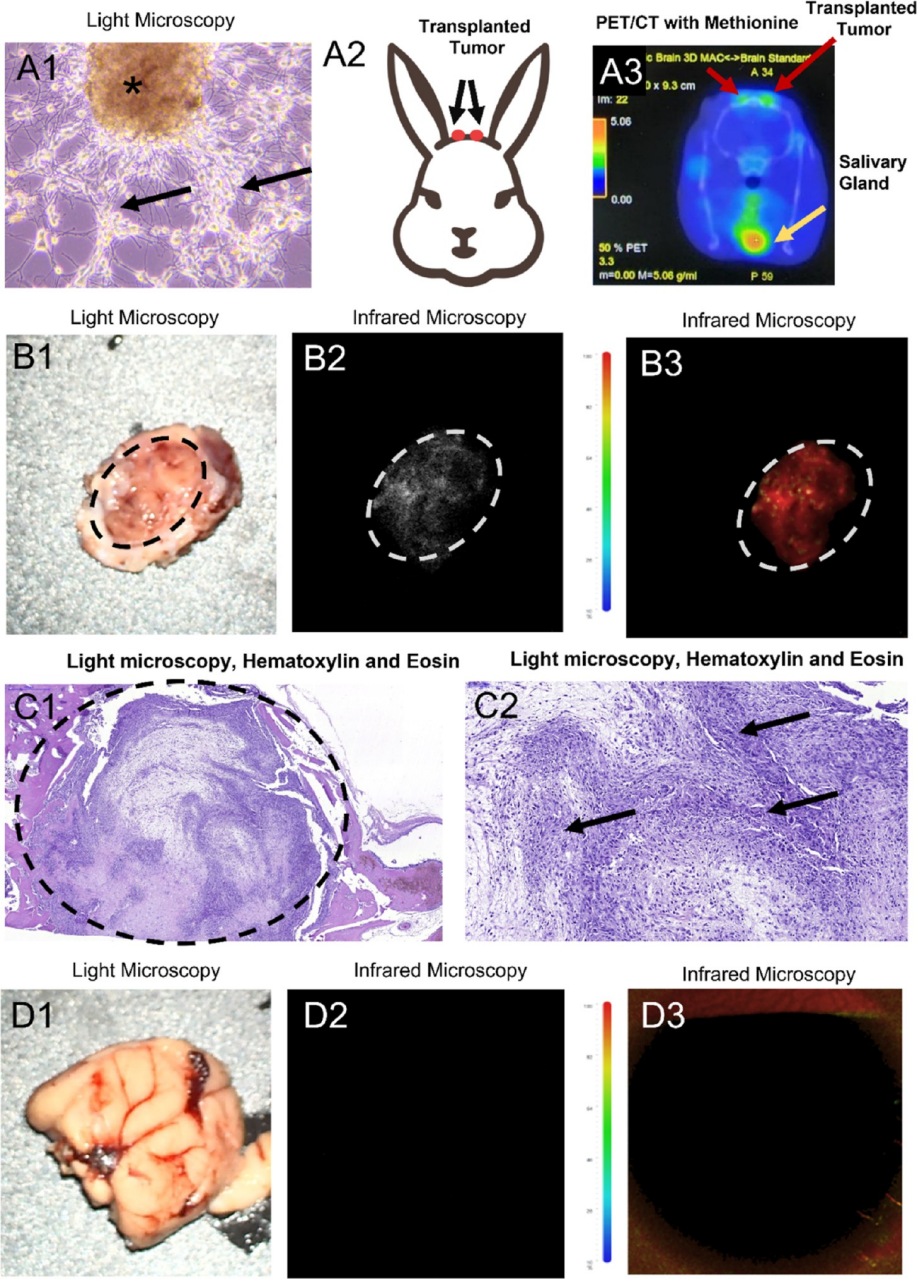
Application of IR-Glint for AptaFGS of the glial tumor was demonstrated on orthotopically xenotransplanted glioblastoma in the rabbit’s brain. Primary glial tumor culture (A1) was transplanted into rabbits’ brains through intracranial windows (A2). PET/CT imaging with ^11^C-methionine confirmed the presence of transplanted tumors in a rabbit (A3). Light (B1) and infrared (B2,B3) microscopy of rabbit’s glial tumors after the surface intracranial application of IR-Glint. H&E staining confirmed glial tumor formation in rabbits’ brains (C1,C2). Dashed lines indicate the tumor, arrows—tumor cell clusters, and *—oncosphere. IR-Glint did not interact with a healthy brain: light (D1) and infrared (D2,D3) microscopy of the healthy rabbit’s brain.

The study aimed to verify the potential use of Glint aptamers labeled with an infrared dye as an intraoperative dye for human glial tumors. Here, we used a tumor model developed in rabbits. For this purpose, the rabbits were first subjected to drug-induced immunosuppression, and then primary cultures evolved from the human anaplastic astrocytoma G4, which harbors two heterozygous mutations making it highly aggressive, were transplanted into their brains through intracranial windows (Figures 9A1,A2 and S9A).

To monitor the development of orthotopically xenotransplanted human glioma in the rabbits’ brains, PET/CT imaging with ^11^C-methionine was used (Figure 9A3). The accumulation of ^11^C-methionine was observed in the area of the trepanation openings in the rabbit brain, specifically at both sites of the transplanted glioma (Figure 9A3).

Furthermore, the brain tissue, unaffected by glioma, and the region of the brain with the growing tumor were stained with IR-Glint and fixed in formalin for analysis using a surgical fluorescent microscope (Figure 9B). The analysis revealed the infrared fluorescence of the rabbit tumor under a surgical fluorescent microscope. On the other hand, the unstained brain tissue and healthy brain tissue stained with IR-Glint did not emit fluorescence in this wavelength range (Figure 9D). However, brain tissue with gliomas stained with aptamers exhibited stable fluorescence in the infrared region of the spectrum.

H&E staining was performed to confirm the presence of glial tumor formation in the rabbits’ brains and skulls, and the results were validated (Figure 9C).

The tumor-to-background ratio (TBR) was determined by comparing the level of fluorescence in the tumor focus to the background fluorescence. This was further tested on postoperative rabbit brain tissues with human glioma xenografts. The rabbit brain was stained with IR-Glint at a concentration of 5 μM, and the fluorescence was evaluated using an infrared module of a Zeiss Kinevo 900 surgical microscope. Figure S7 shows the region of the rabbit skull affected by the tumor under visible and infrared fluorescence.

The TBR value was calculated as the average ratio of the fluorescence levels. In the tumor, focus on the adjacent (unstained) tissues using the ZEN software (Carl Zeiss). Therefore, the calculated TBR value was 24.

To estimate the practical utility and convenience of AptaFGS, we used it on rabbits (Figure S9). The procedure is very simple and fast and does not require intravenous administration.

The aptamers Glint2-1 and Glint5-2 exhibited different stability profiles in fresh mouse blood serum (Figure S11). Glint2-1 remained stable for at least 1 h, whereas Glint5-2 began to degrade after 30 min of incubation in blood serum.

Overall, these findings confirm the potential utility of tumor-specific aptamers labeled with an infrared dye as an intraoperative reagent for detecting and visualizing human glioma in a rabbit model. This technique could significantly improve surgical precision and tumor removal in human patients.

## 3 Discussion

Here, we propose a strategy for fluorescence-guided surgery with infrared-labeled aptamers. These tumor-specific aptamers are a good alternative for the visualization of malignant brain tissues, which makes the surgeon more confident during tumor resection. The infrared-labeled aptamers, IR-Glint, have several advantages that make them attractive to neurosurgeons. The high specificity of IR-Glint to glial tumor tissues and the absence of background fluorescence allow for a high-contrast and bright image, distinguishing the tumor tissue from the healthy brain.

During the investigation, new aptamers, Glint2-1 and Glint5-1, were developed. They exhibited a higher degree of binding to glioma cells while demonstrating a lower level of binding to breast cancer cells and cells derived from a healthy mouse brain. Analysis of histological tissue sections proves the aptamer’s specificity for malignant brain cells.

A commercially available infrared fluorophore, Cy7.5, an analogue of the commonly used indocyanine green, was selected for intraoperative staining. A 5 μM 1:1 mixture of Glint2-1 and Glint5-1 with a Cy7.5 label, called IR-Glint, demonstrated an even better tumor-to-background ratio than that of indocyanine green and does not require intravenous administration. IR-Glint can be made by the robotic synthesis of the DNA oligonucleotides and simultaneous chemical modification by Cy7.5, providing an easy approach to creating an intraoperative spray.

Intraoperative staining and surgery were simulated on postoperative tissues and animal models. Healthy mice and mice with orthotopically transplanted human gliomas were used to determine the optimal strategy for administering the aptamers. It was shown that when administered intravenously, the drug was completely eliminated from the mice’s bodies within 24 h. After 90 min, the drug was distributed throughout the body, and after 5 h, it accumulated in the kidneys, liver, and intestines.

When administered intracranially, the aptamers were visualized at the site of application for at least 90 min. IR-Glint was also metabolized in the liver and spleen, filtered by the kidneys, and excreted through the intestines. Even after 2 h, it could still be seen in the brain at the application site, although the fluorescence was weak.

When administered intravenously, mice with orthotopically xenotransplanted gliomas showed a similar distribution of IR-Glint. 40 minutes after drug injection, it could be visualized in the infrared spectra of the brain at the site where the tumor was transplanted. After 120 min, the tumor remained visible, and fluorescence was also visible in the abdominal cavity. The aptamers were accumulated in the liver, gallbladder, kidneys, and intestines.

Thus, renal filtration and biliary excretion are the presumed mechanisms of elimination of IR-Glint in intracranial or intravenous application cases. Both approaches, intracranial or surface application and intravenous administration, present their own set of benefits and drawbacks. For intracranial or surface applications, the advantages include reducing medication dosage, which could lead to decreased toxicity and costs. It also minimizes the risk of the medication failing to penetrate the blood–brain barrier and degradation by nucleases. However, it necessitates additional handling by the surgeon during the procedure and runs the risk of the drug not reaching distant, nonstained “tentacles” of the tumor. On the other hand, intravenous administration has the benefit of staining the entire tumor before surgery, thus reducing the likelihood of missing any cells. Nevertheless, this method poses a risk of aptamer degradation by blood nucleases and increases the drug’s dosage, cost, and potential toxicity.

Currently, there are a few developments regarding the intraoperative staining of gliomas. The most widely used drug in surgical practice is Gliolan, which is based on 5-aminolevulinic acid, a precursor to protoporphyrin IX (PP) in the human body.^20^ The fluorescence spectrum of PP IX is characterized by two peaks at 635 and 710 nm, with excitation occurring at 405 nm. One drawback of the drug is the lack of targeted delivery, which can lead to false-positive and false-negative results and increased background noise due to the accumulation of 5-aminolevulinic acid in areas with increased metabolism.

Indocyanine green accumulates in neoplastic regions due to its enhanced permeability and retention effect. In regions of the normal brain where the endothelium is intact, it stays within the blood vessels and washes away. In tumors with damaged permeable endothelium, this dye penetrates into the surrounding tissue and persists for an extended period.^21^

Another drug, Petuximab-IRDye800CW (NCT02855086), based on the near-infrared dye RDye800CW and recommended by the US Food and Drug Administration, along with an antibody to human epidermal growth factor, can serve as an analogue to IR-Glint. Table 1 provides a comparative characteristic of the known drugs with IR-Glint.

**Table 1:** Comparative Characteristics of Contrasting Agents with Those of IR-Glint

characteristics	IR-Glint	5-aminolevulinic acid^20^	indocyanine green^21^	petuximab-IRDye800CW*^22^
molecular weight	Glint2-1 8.8 kDa	131 Da		150 kDa
	Glint5-1 13.6 kDa			
tumor-targeted mechanism	targeted delivery	metabolism	accumulation due to enhanced vascular permeability	immunology
mode of administration	intracranial/intravenous	oral	intravenous	intravenous
penetrates the BBB	yes	yes	accumulation in areas of blood–brain-barrier breakdown	yes
good bioavailability	yes	yes	yes	yes
specificity	glioma cells	cells with increased metabolism	no	cancer cells with EGF receptor
real-time intraoperation guidance	yes	yes	yes	yes
low background outfall	yes	yes	yes	yes

* EGFR-targeted intraoperative fluorescence imaging detects high-grade glioma with panitumumab-IRDye800 in a phase 1 clinical trial.^22^

The presented findings and comparative analysis of the medications used for intraoperative glioma visualization, as presented in the table, underscore the potential of incorporating IR-Glint alongside the approved drugs. Undoubtedly, the research will persevere, delving into the properties and mechanisms of action of IR-Glint with the aim of seamlessly integrating it into clinical practice and ensuring that patients can reap the benefits of scientific knowledge and advancements.

## 4 Conclusions

The brain application of the aptamer-based formulation allows for noticeable visualization of the tumor, which can significantly improve the surgeon’s task during brain tumor resection. The results of this study confirm the potential of IR-Glint for intraoperative visualization of gliomas, opening up new avenues in the field of neurosurgery and improving the outcomes of surgical treatment for this tumor disease.

## 5 Methods

### 5.1 Molecular Modeling

Secondary structures of the aptamers were predicted using the mFold^23^ program, which considered experimental parameters such as folding temperature and the presence of ions in the solution. Tertiary structures of the aptamers were modeled with SimRNA^24^ and VMD programs.^25,26^ Molecular dynamic simulations of 200 ns were carried out using the GROMACS 2019.8 package.^27^ The Amber14sb force field^28^ and the TIP3P model^29^ for water were used for simulations. The aptamer was solvated in a periodic cubic box of water. The negative charge of the aptamers was neutralized with Na^+^ ions. Additional Na^+^ and Cl^–^ ions were placed in the system to reach the concentration of 0.15 M. MD simulations were performed with the *NPT* (at constant number of particles *N*, pressure *P*, and temperature *T*) ensemble at 310 K and 1 atm (atm) using the velocity-rescaling thermostat^30^ and at 1 bar pressure using the Parrinello–Rahman barostat.^31^ The clustering analysis of the obtained trajectories was performed using the quality threshold algorithm implemented in the VMD program.^32^

### 5.2 Patient-Derived Tumor Samples

The research we conducted obtained ethical approval from the Local Committee on Ethics in Krasnoyarsk Inter-District Ambulance Hospital, named after N.S. Karpovich, Krasnoyarsk, Russia (Approval #20/11/2016). To collect tumor tissues, we selected patients with glioma who had previously undergone complete curative resection of their disease at Krasnoyarsk Inter-District Ambulance Hospital named after N.S. Karpovich. Prior to the specimens being obtained, written informed consent was obtained from all patients, ensuring their commitment to participate in the study. The solid tumors were handled with the utmost care, maintaining aseptic conditions and immediately placed in an ice-cold colorless DMEM medium supplemented with 1000 U mL^–1^ penicillin G and 1000 mg L^–1^ streptomycin. The samples were then transported on ice to the laboratory within 2–4 h after resection to ensure their viability and optimal condition for subsequent analysis.

### 5.3 Cell Isolation and Culturing

Primary cultures of human brain tumors or breast cancer were obtained from postoperative material. The tumor tissues, after surgical resections, were placed in a sterile 15 mL Falcon tube with 5 mL of cold Hank’s balanced salt solution (HBSS) containing 10% antibiotic–antimycotic.

In a laminar flow hood, the excess Hank’s solution was removed from the tube containing the glial tumor by a dispenser. The tissues were washed three times with 5 mL of cold DPBS to remove blood cells and transferred to a Petri dish filled with 1–2 mL of cold DPBS. Necrotic tissues and blood clots were removed using forceps and a scalpel blade. The remaining tissues were minced into 1 mm^3^ pieces and placed in culture flasks with a nutrient medium for spheroid formation (for glial cells only) or minced into a suspension and filtered through a 70 μm cell strainer into a sterile 15 mL centrifuge tube, followed twice washing by centrifugation at 2000 rpm for 5 min with DPBS.

The resulting pellet, suspended in 2 mL of DPBS, was layered on lymphocytes separation media (3 mL) and centrifuged for 10 min at 2000 rpm. Thereafter, a “cloud” of cells at the border of lymphocyte separation media and DPBS was collected and transferred to a sterile tube with DPBS followed by centrifugation for 5 min at 2000 rpm. The pellet was transferred to a culture flask or a nutrient medium. Cultivation was maintained in a 5% atmosphere of CO_2_ at 37 °C.

To remove cells, the culture was washed with DPBS without Ca^2+^ and Mg^2+^, then poured with 3–5 mL of fresh DPBS solution and held for 2–3 min. The cells were removed with a jet of solution using a dispenser. To separate the attached cells, 2–4 mL of Versen’s solution was poured into the flasks and incubated for 5–15 min. Next, the cell suspension was centrifuged for 3 min at 2500 rpm and washed with DPBS containing Ca^2+^, Mg^2+^.

### 5.4 Flow Cytometric Aptamer Affinity Analysis

Aptamers were synthesized by LLC Lumiprobe (Russia) using the standard phosphoramidite (aminophosphate) method. For the flow cytometry analyses, we used FAM-labeled aptamers. DNA aptamer sequences are given below:

Gli233: 5′-ACT ATT CCA CTG CAA CAA CTG AAC GGA CTG GAA-3′.

Glint2–1: 5′-TTC CAC TGC AAC AAC TGA ACG GAC TGG AA-3′.

Glint2–2: 5′-ATT CCA CTG CAA CAA CTG AAC GGA CTG GAA T-3′.

Gli55: 5′-GTC CGG TTC ACC TCT AGC ATT CCT GGC GTT ATT AAC GGA GCA GTC CTG TGG AGT GGG TGA-3′.

Glint5–1: 5′-CTA GCA TTC CTG GCG TTA TTA ACG GAG CAG TCC TGT GGA GTG GG-3′.

Glint5–2: 5′-TGG CGT TAT TAA CGG AGC AGT CCT GTG GAG TGG GT-3′.

Briefly, glioma primary cell cultures from 5 different patients, breast cancer primary cell cultures from 2 different patients, or brain cells isolated from one healthy mouse were incubated with yeast RNA (1 ng μL^–1^) in 300 μL of DPBS for 30 min at room temperature in a shaker to reduce nonspecific binding. Thereafter, the samples were incubated with 20 nM of FAM-labeled aptamers, FAM-ssDNA library, or FAM-nonspecific randomized sequence as a control for 30 min at room temperature in a shaker. Three technical replicates were done for each cell culture. The affinity of aptamers was measured by flow cytometry using an FC-500 flow cytometer (Beckman Coulter, Inc., USA). Using a blue laser and an FL1 detector capable of registering FAM. The data were analyzed with the help of Kaluza 2.1 software (Beckman Coulter, Inc., USA).

### 5.5 Staining of Postoperative Glioma Tissues

Staining of postoperative glioma samples was performed by using the Cy7.5-labeled aptamers Glint2-1 and Glint5-1. Glioma tissues were obtained from surgical resection, washed with phosphate buffer, and treated with aptamers. Thereafter, the tissues were incubated for 2 min, washed with DPBS, and analyzed using a fluorescent operative microscope, the Zeiss Kinevo 900 (Carl Zeiss, Germany), with the infrared module IR800. The data were analyzed by ZEN 2011 (Carl Zeiss).

### 5.6 Orthotopic Xenotransplantation of Human Glial Tumors

This study was conducted in strict accordance with the National Institute of Health Guidelines’ recommendations for the care and use of laboratory animals. The protocol was approved by the Local Committee on the Ethics of Experiments on Animals of the Krasnoyarsk State Medical University (number #95/2020 from January 29, 2020). All operations were performed under anesthesia, and every effort was made to minimize the animals’ suffering.

#### 5.6.1 Mouse Model

Six-week-old laboratory male ICR mice weighing 28–33 g were maintained in sterile, individually ventilated cages. Mice were immunosuppressed using cyclosporine (20 mg/kg subcutaneously), cyclophosphamide (60 mg/kg subcutaneously), and ketoconazole (10 mg/kg orally) 7 days before and 2 days after transplantation.^22^

Under the inhalation anesthesia, mice hair was removed with hair removal cream, the skin was dissected in a sterile condition, and the cranial window was made using an electro trypan. The formation of the human glial tumor model was carried out by the intracranial injection of primary patient-derived cell cultures. One 1 mm oncosphere and 106 tumor stroma cells in 6 μL of the hydrogel medium (GrowDex/DMEM, 1:1) were placed into a Hamilton syringe between 2 μL of hydrogel medium (GrowDex/DMEM, 2:1). Tumor cells were inoculated into the mice brain through the 2 mm cranial window, the puncture was covered with 5 μL of hydrogel medium (GrowDex/DMEM, 2:1), and the skin incision was sutured.

#### 5.6.2 Rabbit Model

The work was performed on 4 male rabbits (silver breed), weighing 1800–2000 g, with a health certificate. Rabbits were immunosuppressed with cyclosporine (20 mg/kg intramuscularly), cyclophosphamide (20 mg/kg intramuscularly), and ketoconazole (5 mg/kg orally with water) every second day for 21 days before and 2 days after tumor transplantation. Anesthesia in rabbits was carried out with a single intravenous injection of Zoletil-100 solution (manufactured by Virbac, France) at the rate of 0.05 mL per kg of body weight of the experimental animal in combination with the drug Xylavet (manufactured in Hungary) at the rate of 0.15 mL per kg of body weight of the experimental animal. Intravenous administration of drugs in rabbits was carried out after catheterization of the veins on the dorsal surface of the ear with a 24G catheter (mainly the large ear vein was used for catheterization). After reaching the stage of surgical anesthesia, hair was removed from the rabbits using shaving cream, the skin was dissected in the area of the sagittal cross (or temporal line), which must be confirmed with doctors under sterile conditions, and 2 cranial windows with a diameter of 3 mm were made using an electro trypan. The formation of a human glial tumor model was carried out by stereotactic transplantation into the right and left hemispheres of the brain of primary cell cultures of a patient with a glial brain tumor [2 × 10^6^ cells and 5 oncospheres in 40 μL of GrowDex/DMEM medium (1:1)] using a syringe Hamilton with a volume of 40 μL into each cranial window with a diameter of 3 mm. The injection site was covered with skin, and sutures were placed. To avoid the development of a bacterial infection, the animals were intravenously administered the antibacterial drug “Tylosin 50” (manufactured by Nita-Pharm, Russia) within 3 days after surgery.

### 5.7 In Vivo Fluorescence Visualization of IR-Glint in Mice

To determine the optimal strategy for administering IR-Glint (5 μM 1:1 mixture of Glint2-1 and Glint5-1 with a Cy7.5 label) and its distribution, we used healthy mice and mice with human orthotopically transplanted gliomas.

IR-Glint or nonspecific oligonucleotide (50 μL, 5 μM) was administered to healthy mice, healthy mice with trepanation holes in the skull, and mice with transplanted glial tumors by intravenous injection or surface application (subcutaneous injection into mice with a trepanation hole). Intact noninjected mice with the transplanted glial tumors were taken as control ones. IR fluorescence in mice under inhalation anesthesia was evaluated by using a Fluor i In Vivo imaging system (South Korea) with a red excitation laser and infrared-emitting filter with the same settings for all experimental mice. All experiments were performed in three replicates.

### 5.8 AptaFGS

Glial tumor staining in mice was performed by intravenous injection or direct staining by spraying a 5 μM 1:1 mixture of Glint2-1 and Glint5-1 with a Cy7.5 label. In the first case, 200 μL of aptamers were injected into a tail vein for 30 min before brain removal. In the second case, 200 μL of aptamers were powered on the mouse brain under anesthesia and incubated for 3 min.

Mice organs were put in 10% formalin and then analyzed with the help of an operative microscope with an IR800 Zeiss Kinevo 900 infrared module (Carl Zeiss, Germany).

Intraoperative staining of the glioma in rabbits was performed using pooled Glint2-1 and Glint5-1 labeled with Cy7.5 (IR-Glint) at a concentration of 5 μM. Anesthesia was induced using Zoletil-100 solution (manufactured by Virbac, France) at the rate of 0.05 mL per kg of body weight of the experimental animal in combination with the drug Xylavet (manufactured in Hungary) at the rate of 0.15 mL per kg of body weight of the experimental animal. After surgery and staining, an excess amount of Zoletil was administered to the animal, and the brain was removed. The tissues were fixed in 10% neutral formalin and then analyzed using a surgical fluorescence microscope with an IR800 Zeiss Kinevo 900 infrared module (Carl Zeiss, Germany).

### 5.9 Toxicity Testing of Aptamers in Mice

To determine toxicity, changes in cholesterol, serum alanine aminotransferase (ALT), alkaline phosphatase (ALP), bilirubin, total protein, and alpha-amylase were evaluated.

Hepatotoxicity assessment was carried out in healthy male and female mice injected with 200 mL of 5 μM IR-Glint into tail veins. One week after the injection, blood was harvested and submitted to a clinical laboratory for analysis.

### 5.10 Evaluation of Aptamers Stability in Blood Serum

The stability of the aptamers Glint2-1 and Glint5-1 in undiluted fresh mouse blood serum was assessed through 2% agarose horizontal gel electrophoresis. A 5 μL FAM-labeled Glint2-1 or Glint5-1 was mixed with undiluted blood serum and, after incubation at 37 °C for 0, 5, 30, 60, 120 min, and 24 h, was loaded into the wells on the agarose gel. The intact FAM-labeled Glint2-1 and Glint5-1 aptamers in PBS were the reference molecules. The gel was then subjected to electrophoresis in a gel electrophoresis system (Advance Mupid-One, Belgium) for 20 min at 100 V, and the fluorescence levels were analyzed using a fluorescent gel documentation system G-Box (Syngene, Cambridge, UK). Degradation was assessed by monitoring the decrease in the band intensity over time in mouse blood serum.

### 5.11 In Vivo Visualization of Human Glioma Xenotransplantation in a Rabbit Using PET/CT

Tumor location in rabbits was monitored using PET/CT. Rabbits were injected into an ear vein with ^11^C methionine for 40 min before visualization. The animals were immobilized using a single intravenous injection of Zoletil-100 solution (manufactured by Virbac, France) at the rate of 0.05 mL per kg of body weight of the experimental animal in combination with the drug Xylavet (manufactured in Hungary) at the rate of 0.15 mL per kg of body weight of the experimental animal. PET/CT scanning was performed using a Discovery PET/CT 600 scanner (General Electric, USA). Data were analyzed using PET VV software at an AW Volume Share 5 workstation and Hounsfield densitometry scale.

### 5.12 Histological and Confocal Laser Scanning Microscopy Analysis of Tissues

Human postoperative tissues were incubated with 1 ng mL^–1^ yeast RNA for 30 min on a shaker. Thereafter, it was washed two times and incubated with 50 nM FAM-labeled Glint2-1 or Glint5-1 for 30 min on a shaker and fixed at 10% buffered formalin solution. The volume of a fixative was 10 times greater than the size of the immersed tissue. The samples underwent a standard histological treatment based on isopropyl alcohol with further paraffin impregnation. Sections made from paraffin blocks were applied to positively charged adhesive glasses. One part of the section was left for confocal microscopy analysis without additional staining; the next one was stained with hematoxylin and eosin dyes to confirm tissue morphology.

Histological tissue sections of mice and rabbit brains were fixed in a formalin solution stained with hematoxylin and eosin dyes to confirm the tissue morphology.

A Nexcope NIB900 (Ningbo Yongxin Optics Co., Ltd., China) and LSM 780 NLO Confocal microscope (Carl Zeiss, Germany) were used for confocal imaging; images were processed with ZEN2 software.

### 5.13 Statistical Analyses

Blood serum biochemical parameters were compared using an ANOVA. A two-tailed *t*-test was used to compare the group means. A Bonferroni correction was applied to the *p* values. The differences were considered significant at a level of significance of *p* ≤ 0.05.

### 5.14 Molecular Profiling

The PCR assays were performed using Readymix Screenmix (Evrogen, Russia) in a total volume of 25 μL. Primers used for PCR and cycle sequencing are presented in Table 2. PCR amplicons were purified by the Biolabmix Kit (Biolabmix, Russia) to isolate DNA and RNA from the reaction mix.

**Table 2:** Primers Used for PCR and Cycle Sequencing

name	primer sequence (5′–3′)	fragment length
IDH1_p.R132_F	CACCAAATGGCACCATACGA	311 bp
IDH1_p.R132_R	ATGTGTTGAGATGGACGCCT	
IDH2_p.R140Q _F	TCTGCCACAAAGTCTGTGGCCTTGT	317 bp
IDH2_p.R140Q_R	AAAGATGGCGGCTGCAGTGGG	
TP53_p.R175_F	TCAACTCTGTCTCCTTCCTC	328 bp
TP53_p.R175_R	TCCACTCGGATAAGATGCTG	
TP53_p.R248Q_F	AATGGCGTGAACCTGGGCGGT	375 bp
TP53_p.R248Q_R	GAAATCGGTAAGAGGTGGGC	
TP53_p.R273_F	CAAGGGTGGTTGGGAGTAGA	243 bp
TP53_p.R273_R	GCTTACCTCGCTTAGTGCTC	

Sanger sequencing was performed using Nimagen BrilliantDye Terminator (v1.1) chemistry and an Applied Biosystems 3500 genetic analyzer based on capillary electrophoresis (Thermo Fisher Scientific, USA). Sequencing products were purified with the D-pure DyeTerminator Cleanup Kit (Nimagen, Netherlands), and sequences were assembled with SnapGene software (Dotmatics, USA).

## Supporting Information Available

Additional figures about fluorescent images of IR-Glint in the solution of DPBS and glial tumor tissues; distribution of IR-Glint in healthy mice after intravenous injection and subcutaneous injection; infrared background fluorescence of noninjected mice; distribution of Cy7.5-labeled nonspecific oligonucleotide in mice; accumulation of indocyanine green in brain tumor; background IR fluorescence of orthotopically xenotransplanted glioblastoma in mice; blood biochemical parameters of mice injected with IR-Glint; in vivo visualization of human xenotransplantated glioma and AptaFGS in a rabbit; region of the rabbit skull affected by glioma is shown in visible light and infrared fluorescence; and stability of the IR-Glint aptamers in mice blood serum (PDF)
