# Supplementary material for: Visualization of Brain Tumors with Infrared-Labeled Aptamers for Fluorescence-Guided Surgery
Source: J Am Chem Soc. 2024 Aug 26;146(36):24989–5004. doi: 10.1021/jacs.4c06716 (PMC11404482; doi:10.1021/jacs.4c06716)
Supplement: Supplementary file 1 — ja4c06716_si_001.pdf [file ja4c06716_si_001.pdf]

Supporting Information

Visualization of Brain Tumors with Infrared-Labeled Aptamers for Fluorescence-Guided Surgery

Galina Zamay[1,2,3], Anastasia Koshmanova[1], Andrey Narodov[1,2], Anton Gorbushin[1,2,4], Ivan Voronkovskii[1,2,4], Daniil Grek[1,2], Natalia Luzan[1], Olga Kolovskaya[1,2,3], Irina Shchugoreva[1,3], Polina Artyushenko[1,3], Yury Glazyrin[1,3], Victoriya Fedotovskaya[1,3], Olga Kuziakova[6], Dmitry Veprintsev[3], Kirill Belugin[7], Kirill Lukyanenko[1,3], Elena Nikolaeva[1,3], Andrey Kirichenko[1,2], Ivan Lapin[8], Vladimir Khorzhevskii[1,9], Evgeniy Semichev[1], Alexey Mohov[3], Daria Kirichenko[1], Nikolay Tokarev[7], Natalia Chanchikova[7], Alexey Krat[1,10], Ruslan Zukov[1, 10], Varvara Bakhtina[1], Pavel Shnyakin[1], Pavel Shesternya[1], Felix Tomilin[3,11], Aleksandra Kosinova[1,3], Valery Svetlichnyi[8], Tatiana Zamay[1,3], Vadim Kumeiko[5,6], Vasily Mezko[2], Maxim V. Berezovski[12], and Anna Kichkailo[1,2,3]**

[1]Prof. V.F. Voino-Yasenetsky Krasnoyarsk State Medical University, Krasnoyarsk, 660022, Russia

[2]Aptamerlab LLC, Krasnoyarsk, 660042, Russia

[3]Federal Research Center "Krasnoyarsk Science Center of the Siberian Branch of the Russian Academy of Sciences," Krasnoyarsk 660036, Russia

[4]Krasnoyarsk Inter-District Ambulance Hospital named after N.S. Karpovich, 17 Kurchatova, Krasnoyarsk 660062, Russia

[5]A.V. Zhirmunsky National Scientific Center of Marine Biology, Far Eastern Branch of Russian Academy of Sciences, Vladivostok, 690041, Russia.

[6]School of Medicine and Life Sciences, Far Eastern Federal University, Vladivostok, 690922, Russia

[7]Federal Siberian Research Clinical Centre under the Federal Medical Biological Agency, Krasnoyarsk, 660130, Russia

[8]Laboratory of Advanced Materials and Technology, Tomsk State University, Tomsk, 634050, Russia

[9]Krasnoyarsk Regional Pathology-Anatomic Bureau, Partizana Zheleznyaka, Krasnoyarsk 660022, Russia

[10]Krasnoyarsk Regional Clinical Cancer Center, 16 1-ya Smolenskaya, Krasnoyarsk 660133, Russia

[11]Kirensky Institute of Physics, 660012, Russia

[12]Department of Chemistry and Biomolecular Sciences, University of Ottawa, Ottawa, Ontario K1N 6N5, Canada

Corresponding authors: Maxim V. Berezovski (maxim.berezovski@uottawa.ca) and Anna Kichkailo (annazamay@yandex.ru)

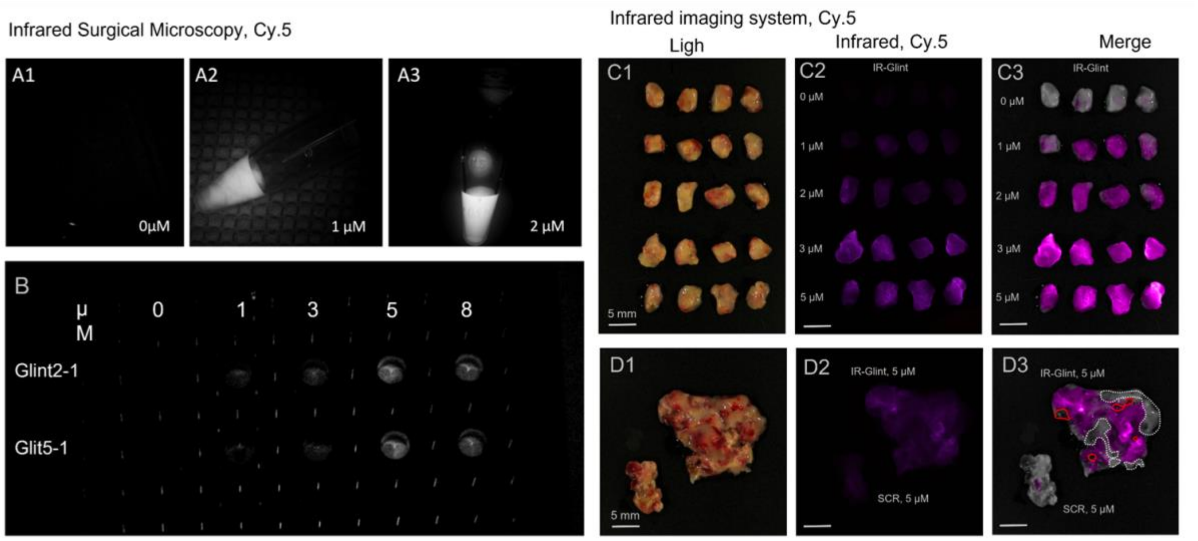

Figure S1. Fluorescent microscopy of IR-Glint (A) in the solution of DPBS in concentrations 0 µM (1), 1 µM (2), and 2 µM (3). Panel B demonstrates glial tumor tissues incubated with Cy 7.5-labeled aptamers (Glint2-1 and Glint5-1) in 1 – 8 µM under the IR module of the surgical microscope Zeiss Kinevo 900. In panel A, the IR-Glint was in 1.5 ml vials. In panel B, glial tumor tissue samples were in a 96-well plate. Detailed analyses of glial tumor tissue staining were performed using an infrared imaging system in four repetitions (C). Comparative imaging of a scrambled sequence (SCR) stained a small tumor piece and IR-Glint stained a large glial tumor (D). The white dashed line indicates necrotic zones, and the red line indicates blood clots.

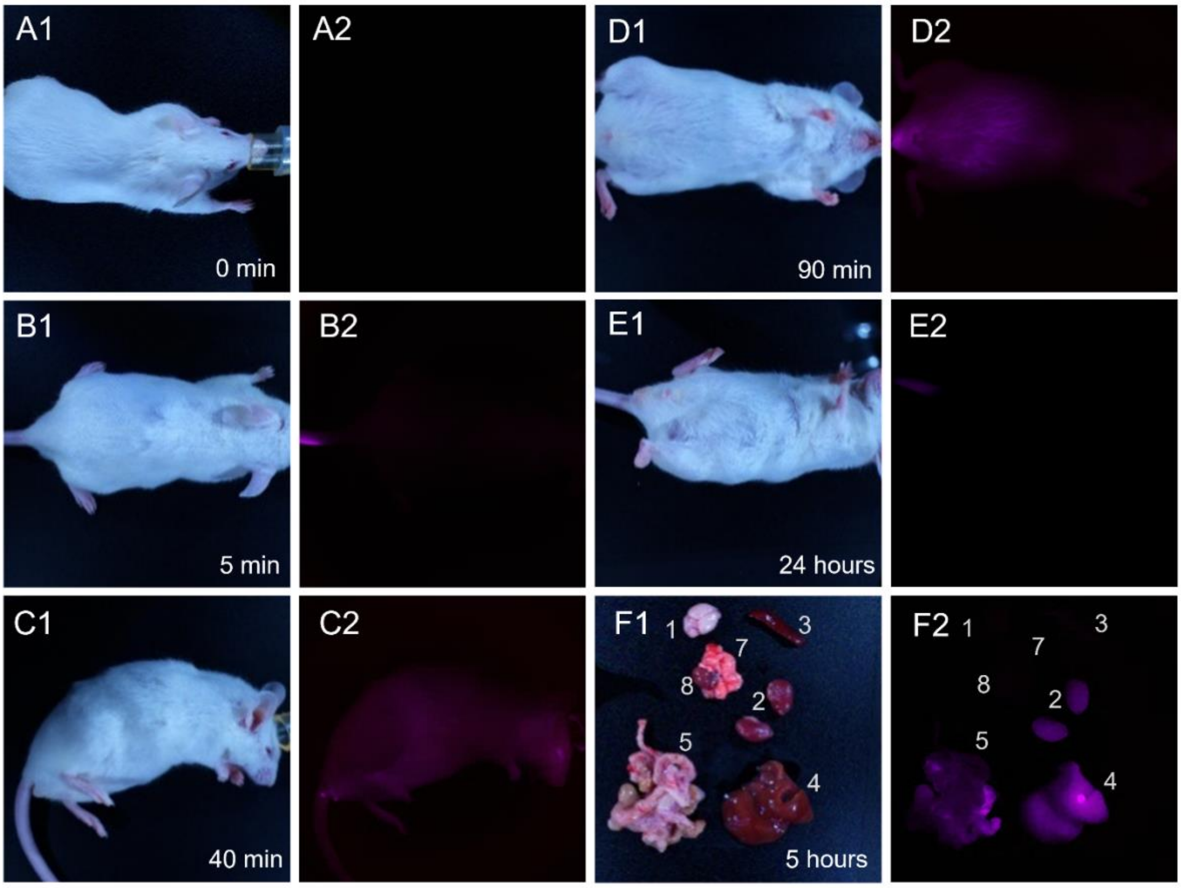

Figure S2. Distribution of IR-Glint in healthy mice after intravenous injection registered at Brightfield (1), IR fluorescence (F). Mouse prior injection (A), 5 minutes (B), 40 minutes (C), 90 minutes (D), and 24 hours (E) after the tail vein injection. Accumulation of IR-Glint in organs (F): 1 - brain, 2 – kidneys, 3 - spleen, 4 - liver, 5 - intestines, 6 - gallbladder, 7 - lungs, and 8 - heart.

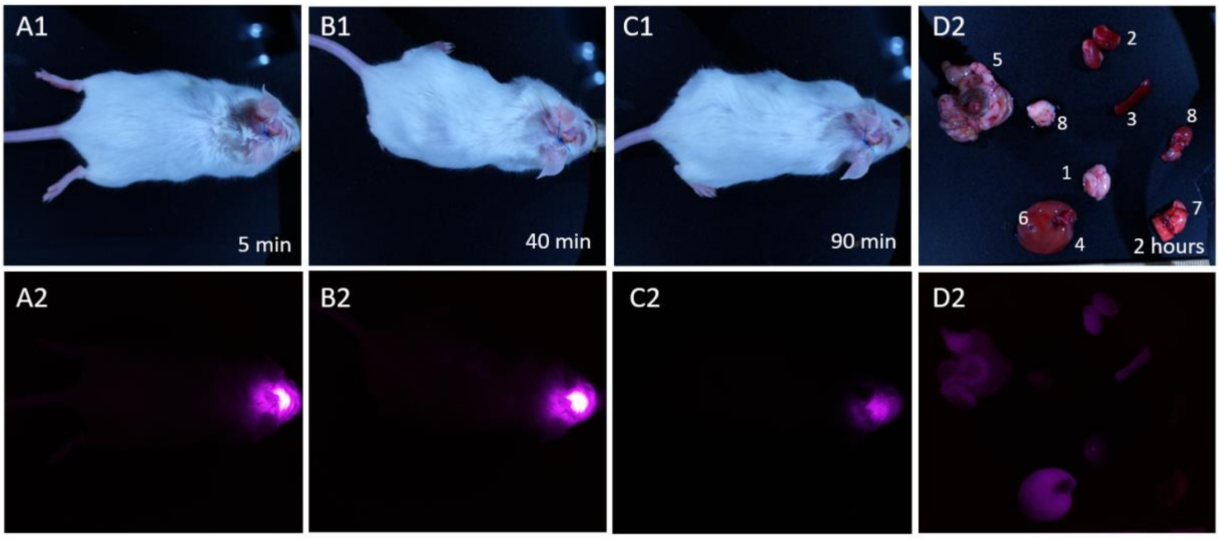

Figure S3. Distribution of IR-Glint in healthy mice with intracranial window after subcutaneous injection registered at Brightfield (1), IR fluorescence (2). Mouse after 5 minutes (A), 40 minutes (B), and 90 minutes (C) of the subcutaneous injection. Accumulation of IR-Glint in organs (D): 1 – brain, 2 – kidneys, 3 – spleen, 4 – liver, 5 – intestines, 6 – gallbladder, 7 – lungs, and 8 – heart, 9 – peritoneal fat.

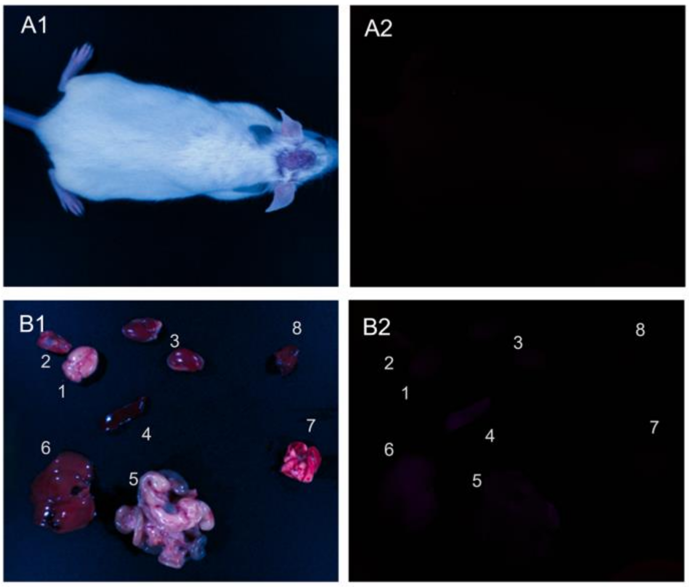

Figure S4. Infrared background fluorescence of non-injected mouse. Brightfield (1), IR fluorescence (2). Intact mouse (A) with the transplanted tumor and organs (B): 1 – brain, 2 – tumor inside the skull, 3 – kidneys, 4 – spleen, 5 – intestines, 6 – liver, 7 – lungs, and 8 – heart.

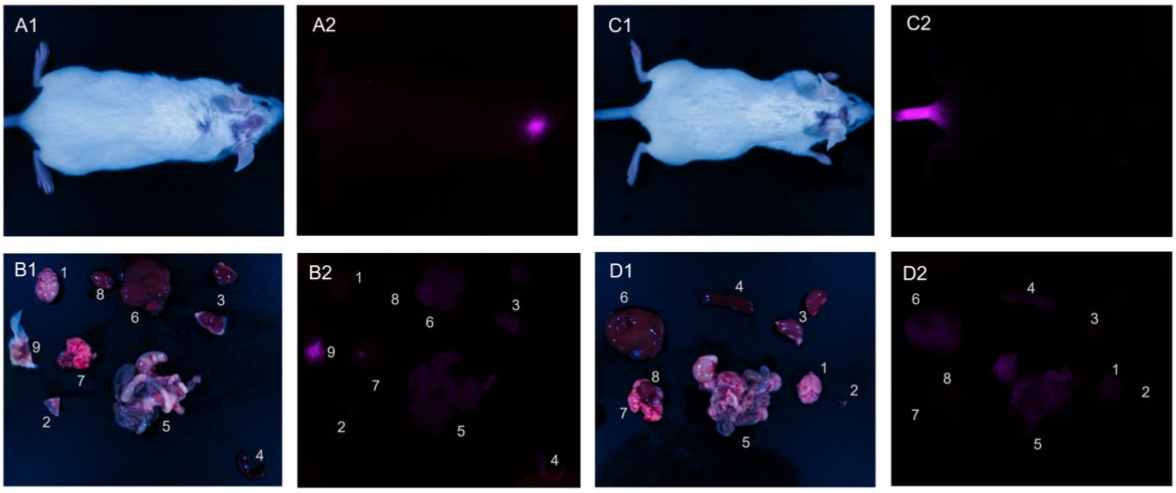

Figure S5. Distribution of Cy7.5-labeled non-specific oligonucleotide in mice with glial tumor transplanted through the intracranial window. Mice after 90 minutes of subcutaneous (A) and tail vein injection (C) of Cy7.5-labeled non-specific oligonucleotide, registered at brightfield (1) and IR fluorescence (2). Accumulation of Cy7.5-labeled non-specific oligonucleotide in organs 5 hours after subcutaneous (B) and tail vein (D) injection: 1 – brain, 2 – kidneys, 3 – spleen, 4 – liver, 5 – intestines, 6 – gallbladder, 7 – lungs, and 8 – heart, 9 – peritoneal fat.

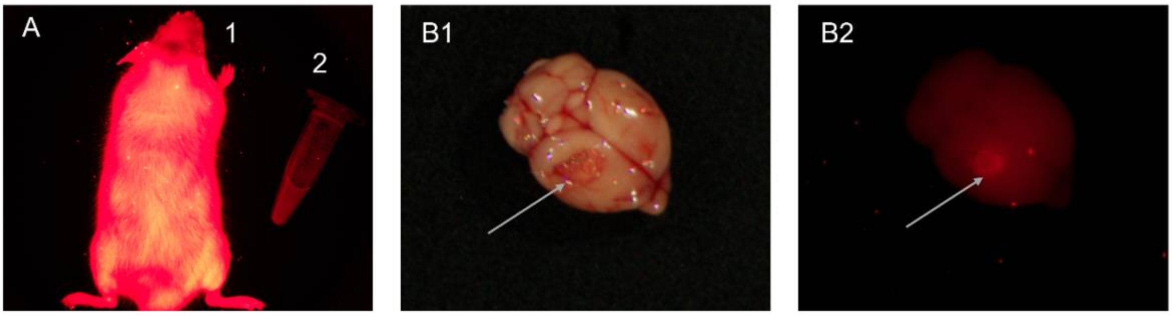

Figure S6. Accumulation of indocyanine green in mouse brain tumor. The background autofluorescence of mice skin (A, 1) and ingocyane green fluorescence in the tube (A, 2). Ingocyane green accumulates in the tumor (B, arrow) 2 hours after tail vein injection. Brightfield (B1), IR fluorescence (B2).

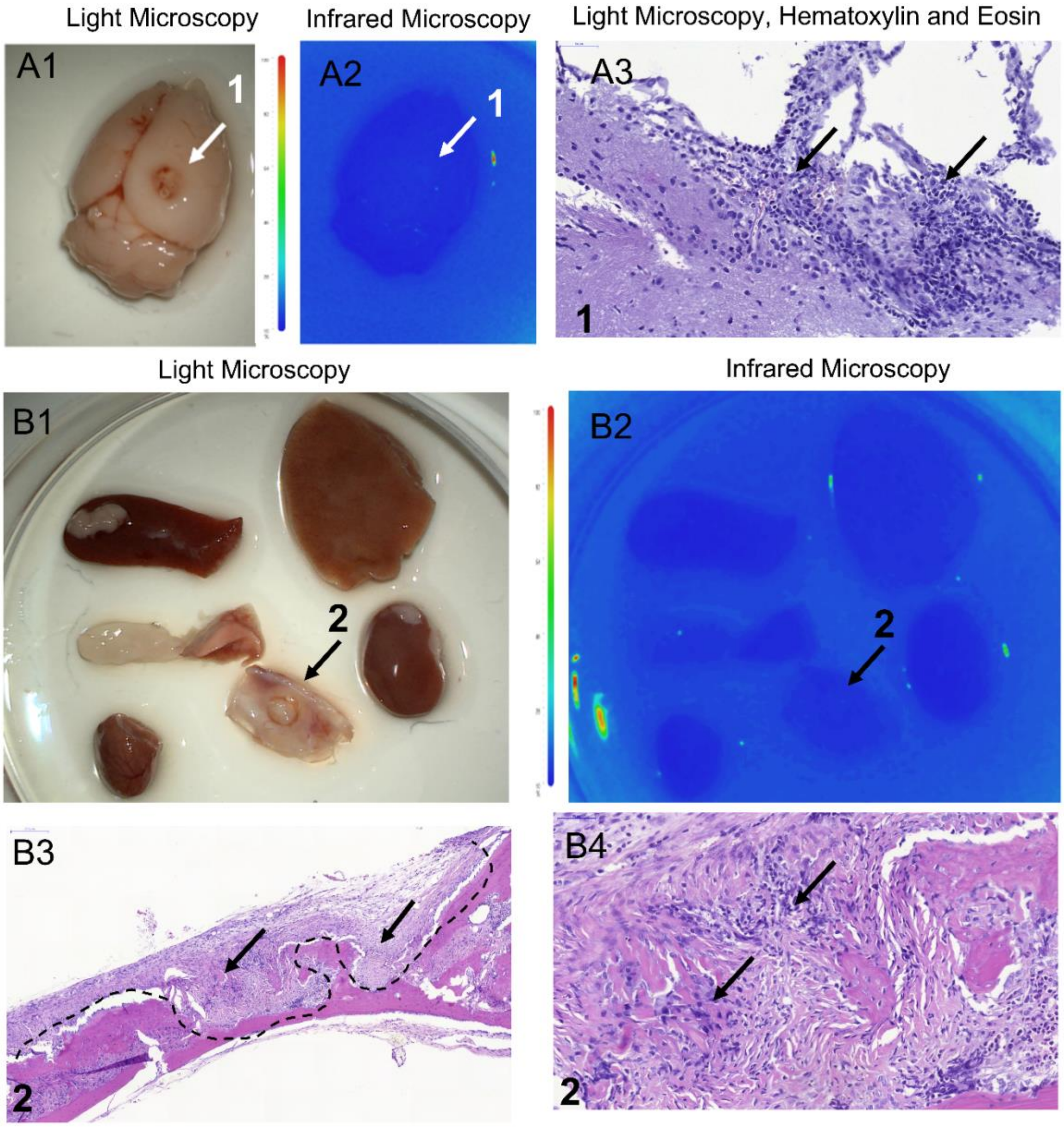

Figure S7. Background IR fluorescence (A2, B2) and light microscopy (A1, B1) of orthotopically xenotransplanted glioblastoma (A1, arrow 1), tumor inside the skull (B1, arrow 2) and organs (B1) without IR-Glint administration. H&E staining confirmed glial tumor formation in mice brains (A3) and inside the skull (B3, B4).

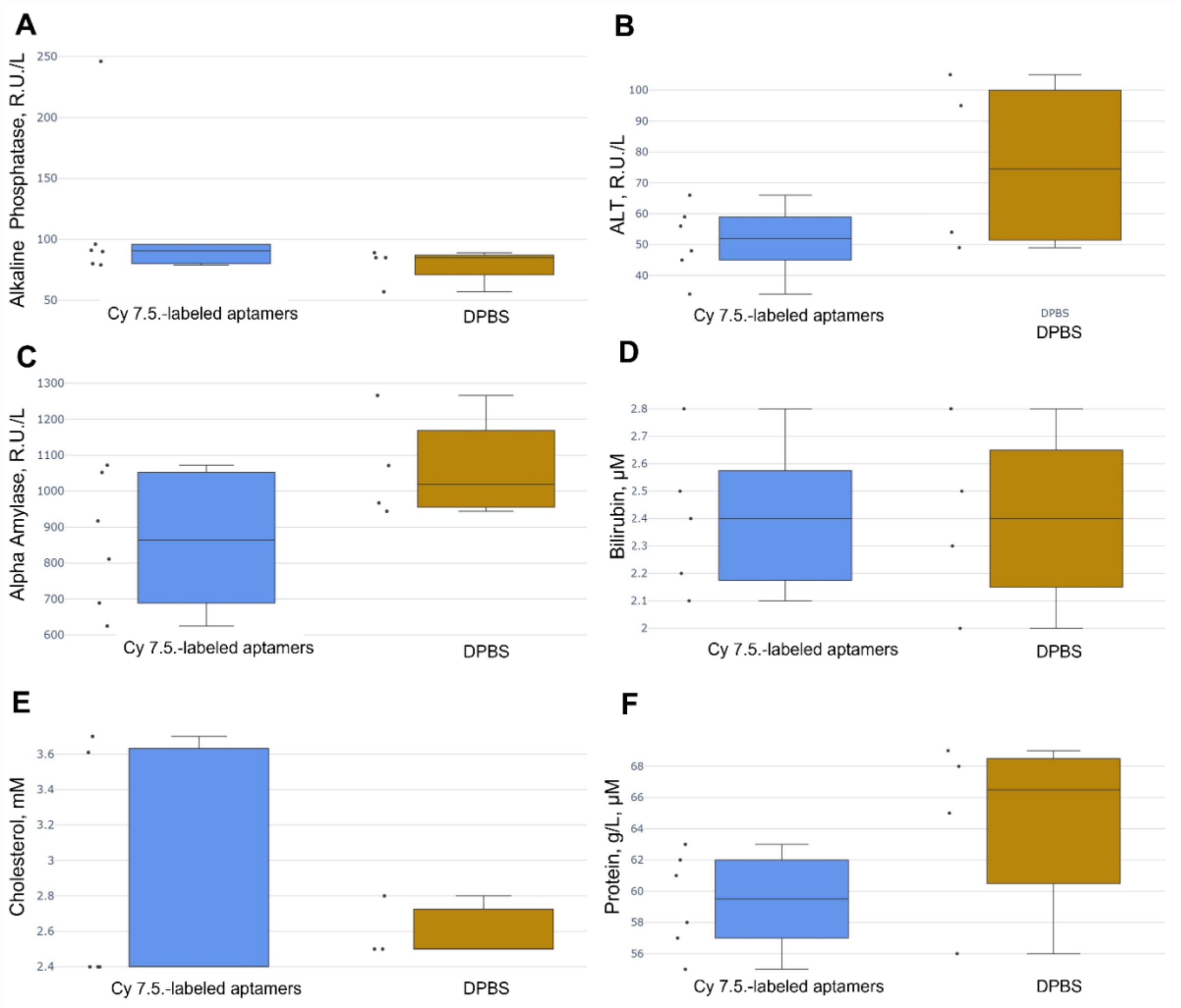

Figure S8. Blood biochemical parameters of mice injected with IR-Glint.

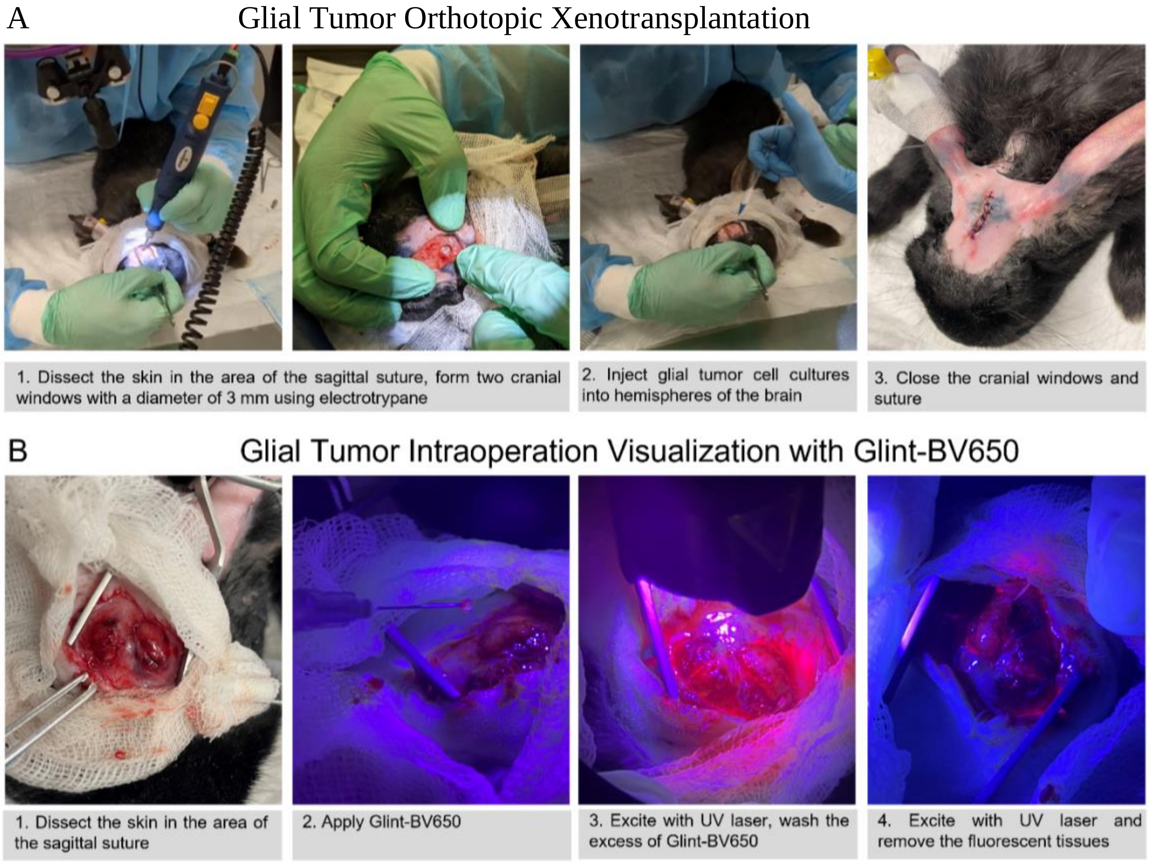

Figure S9. In vivo visualization of human xenotransplantated glioma and AptaFGS modeling in a rabbit. Glial tumor orthotopic xenotransplantation (A). Glial tumor intraoperative visualization (B).

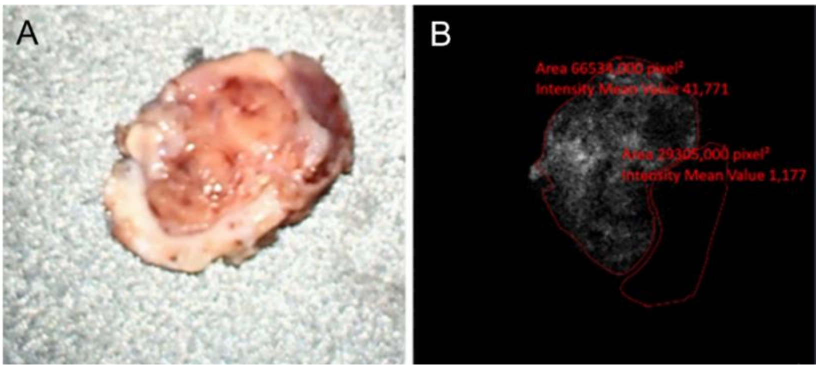

Figure S10. The region of the rabbit skull affected by glioma is shown in visible light (A) and infrared fluorescence (B).

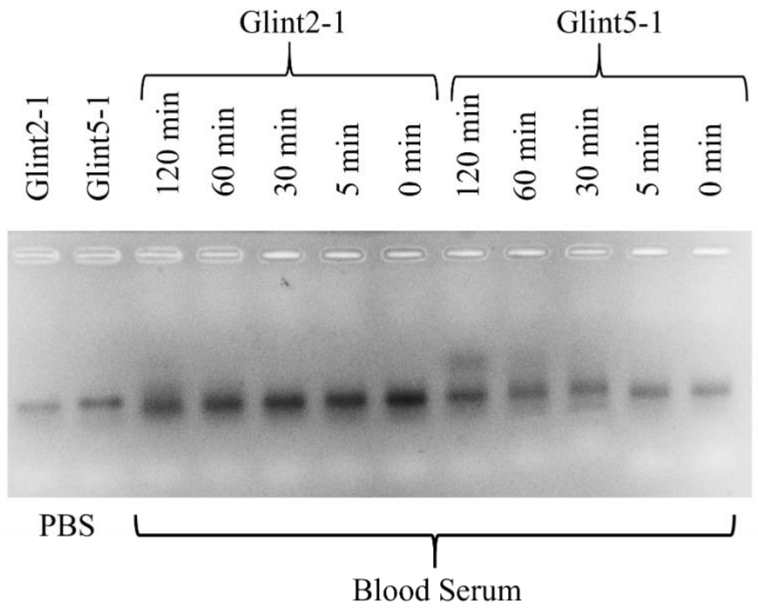

Figure S11. Stability of the IR-Glint aptamers (Glint2-1 and Glint5-1) in mouse blood serum.
